# Comparative Lipidomic Analysis of Extracellular Vesicles Derived from *Lactobacillus plantarum* APsulloc 331261 Living in Green Tea Leaves Using Liquid Chromatography-Mass Spectrometry

**DOI:** 10.3390/ijms21218076

**Published:** 2020-10-29

**Authors:** Hyoseon Kim, Minjung Kim, Kilsun Myoung, Wanil Kim, Jaeyoung Ko, Kwang Pyo Kim, Eun-Gyung Cho

**Affiliations:** 1 Department of Applied Chemistry, Institute of Natural Science, Global Center for Pharmaceutical Ingredient Materials, Kyung Hee University, Yongin 17104, Korea; invaluably@naver.com (H.K.); tuxmfdyd1@daum.net (M.K.); 2 Basic Research and Innovation Division, R&D Center, Amorepacific Corporation, Yongin 17074, Korea; ksmyoung@amorepacific.com (K.M.); wkim83@gmail.com (W.K.); jaeyoungko@amorepacific.com (J.K.); 3 Division of Cosmetic Science & Technology, Daegu Haany University, Gyeongsan 38610, Korea; 4 Department of Biomedical Science and Technology, Kyung Hee Medical Science Research Institute, Kyung Hee University, Seoul 02453, Korea

**Keywords:** *Lactobacillus plantarum* APsulloc 331261, green tea leaf, extracellular vesicles, lipidomic analysis, liquid chromatography-mass spectrometry, intercellular communication

## Abstract

*Lactobacillus plantarum* is a popular probiotic species due to its safe and beneficial effects on humans; therefore, novel *L. plantarum* strains have been isolated and identified from various dietary products. Given that bacteria-derived extracellular vesicles (EVs) have been considered as efficient carriers of bioactive materials and shown to evoke cellular responses effectively, *L. plantarum*-derived EVs are expected to efficiently elicit health benefits. Herein, we identified *L. plantarum* APsulloc 331261 living in green tea leaves and isolated EVs from the culture medium. We performed quantitative lipidomic analysis of *L. plantarum* APsulloc 331261 derived EVs (LEVs) using liquid chromatography-mass spectrometry. In comparison to *L. plantarum* APsulloc 331261, in LEVs, 67 of 320 identified lipid species were significantly increased and 19 species were decreased. In particular, lysophosphatidylserine(18:4) and phosphatidylcholine(32:2) were critically increased, showing over 21-fold enrichment in LEVs. In addition, there was a notable difference between LEVs and the parent cells in the composition of phospholipids. Our results suggest that the lipidomic profile of bacteria-derived EVs is different from that of the parent cells in phospholipid content and composition. Given that lipids are important components of EVs, quantitative and comparative analyses of EV lipids may improve our understanding of vesicle biogenesis and lipid-mediated intercellular communication within or between living organisms.

## 1. Introduction

Probiotics, beneficial components of the microbiota, can influence gut microbes which interact with the immune system and broaden the metabolic potential of the host. Inflammatory or metabolic intestinal diseases such as diarrhea, *Helicobacter pylori* infection, inflammatory bowel disease, and type 2 diabetes are often linked to compositional changes in the microbiota [1,2]. When administered in adequate amounts, probiotics can prevent or treat these diseases, providing health benefits to the host by modulating immunological and gastrointestinal functions directly or indirectly (FAO/WHO, 2001). As the largest organ for microbial cells to colonize in the human body, the skin acts as a physical barrier to prevent the invasion of foreign pathogens while providing a home to the commensal microbiota similar to the gut, and some skin diseases are also associated with an altered microbial state [3,4]. Therefore, the restoration of microbial balance in not only the intestine but also the skin using probiotics has drawn public attention as an attractive method to benefit human health.

The most common strains used as probiotics are the lactic acid bacteria (LAB) such as *Lactobacillus* and *Bifidobacterium* [5,6]. These are Gram-positive bacteria marked by their lack of lipopolysaccharides and secreted proteases, and therefore are commonly used as probiotics and delivery vehicles for therapeutic compounds and proteins, or for food production. Among *Lactobacillus* species possessing probiotic properties [7,8,9], *Lactobacillus plantarum* is naturally found in many fermented food products as well as anaerobic plant matter, and possesses a large genome based on completed sequencing [10]. Its flexible and adaptive behavior makes it well-suited to many different environmental niches, a quality attributed to its possession of a relatively large number of regulatory and transport functions [10]. *L. plantarum* can therefore grow at anywhere between 15 and 45 °C and at any pH level of 3.2 or higher, and survives in the human gastrointestinal tract, which is an important aspect in selecting a candidate probiotic strain [11]. The safety and the effects of *L. plantarum* on the host intestinal tract and related molecular studies have been addressed [12,13,14], suggesting it as an effective and beneficial probiotic species. Previously, the strains *L. plantarum* APsulloc were isolated and identified from green tea leaves and of them, the safety and probiotic properties of one strain, *L. plantarum* APsulloc 331261, were evaluated [15,16].

Like mammalian cells, Gram-negative and -positive bacteria release extracellular vesicles (EVs), called outer membrane vesicles and membrane vesicles, respectively, for intercellular and bacteria–host interactions [17,18,19]. As a conserved characteristic with mammalian EVs including exosomes and microvesicles, EVs are a normally spherical structure enclosed by a lipid bilayer with an average diameter of 20–1000 nm. They contain bioactive materials including proteins, lipids, and nucleic acids [20], and are involved in numerous pathophysiological processes. In particular, Gram-positive bacteria-derived EVs are often associated with various virulence factors such as protein A *(Staphylococcus aureus*), pneumolysin (*Streptococcus pneumonia*), and anthrax toxin (*Bacillus anthracis*). EVs are more cytotoxic or effective to cells than disrupted bacterial extracts or purified toxin alone [19,21,22], indicating that EVs are efficient carriers of bioactive materials and evoke cellular responses potently. In contrast with those of virulent bacteria, EVs derived from commensal Gram-positive probiotic strains (probiotic EVs) are considered to efficiently carry bioactive materials important for eliciting therapeutic benefit [23,24,25]. Indeed, we found that *L. plantarum* APsulloc derived EVs (LEVs) exert beneficial effects on human skin by promoting differentiation of human monocytic THP1 cells towards an anti-inflammatory M2 macrophage phenotype, especially M2b [26]. In this regard, the analysis of enriched materials in probiotic EVs and the investigation of molecular mechanisms on how probiotic EVs mediate beneficial effects on the host are necessary to develop EVs as potent probiotic substances to facilitate human health.

Contrary to the proteomic analysis of bacterial EVs, which is generally performed to understand pathogenic regulation mediated by EV-enriched proteins in the host–pathogen interaction, the lipidomics of bacterial EVs and their possible roles are largely unknown. Given that probiotics and their EVs are supposed to lack toxic materials, we assumed that the lipidomic analysis of LEVs and their parent cells may provide alternative information useful for understanding their beneficial effects on the host and for developing a delivery vehicle for therapeutic compounds and proteins, although there is only a limited number of studies related to the mechanisms underlying the biogenesis and lipid composition of EVs in Gram-positive bacteria [27,28].

In this study, for the first time, we present the comparative lipidomic analysis of EVs derived from a probiotic strain *L. plantarum* APsulloc 331261 using liquid chromatography-mass spectrometry (LC-MS). *L. plantarum* APsulloc 331261 was isolated from fermented green tea leaves, and EVs were successfully purified from the culture supernatant of the bacteria. We compare the lipidomic profile of EVs with that of parent cells *L. plantarum* APsulloc 331261, and discuss the meaning of differential expression in lipid classes.

## 2. Results

### 2.1. Strain Isolation and Identification

Fifteen colonies of putative lactic acid bacteria (LAB) were isolated from fermented green tea leaves following the procedure described in Figure S1. In phylogenetic analysis of isolated LAB colonies, nine of them were *Lactobacilli* species and, of the nine, four belonged to *L. plantarum* (Figures S1 and S2). These four strains were named as *L. plantarum* APsulloc with a specific number, in particular, *L. plantarum* APsulloc 331261 was chosen for comparative lipidomic analysis on their EVs and the cells (Figure S2).

### 2.2. Spontaneous Release of EVs from L. plantarum APsulloc 331261

**Figure 1:**
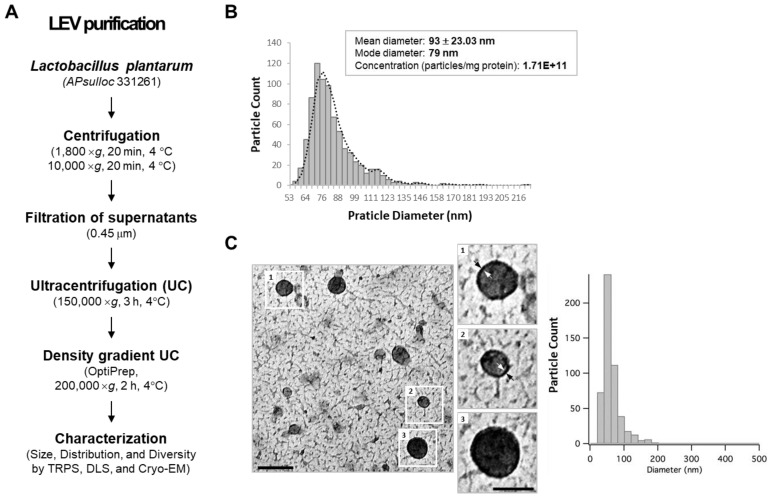
*Lactobacillus plantarum* APsulloc 331261 spontaneously releases extracellular vesicles. (**A**) Scheme for *L. plantarum* APsulloc derived extracellular vesicle (LEV) purification. (**B**) Size distribution, dispersity, mean diameter/mode diameters, and concentration of purified LEVs based on tunable resistive pulse sensing (TRPS) analysis using qNano-Gold. (**C**) Cryo-TEM image of LEVs (left). The outlined LEV images are enlarged and the lipid bilayer indicated by black and white arrows (middle). The size distribution of LEVs was automatically analyzed on cryo-TEM images (right). Scale bars, 200 nm (low) and 100 nm (high magnification).

To examine whether *L. plantarum* APsulloc 331261 (hereafter, referred to as *L. plantarum*) secretes EVs, we isolated EVs from *L. plantarum* culture supernatant using ultracentrifugation and density gradient ultracentrifugation methods according to a previously described isolation method for Gram-positive bacterial EVs (Figure 1A) [17,29]. Based on dynamic light scattering (DLS) and tunable resistive pulse sensing (TRPS) analyses, the size of density-purified, *L. plantarum*-derived EVs (LEVs) was less than 100 nm in diameter with a mean value of 93 ± 23.03 nm. The major populations, however, were distributed between 72 ~ 84 nm, showing broad dispersity in purified LEVs (PDI > 0.4) (Figure 1B). The particle numbers were an average of 1.71 × 10^11^ per mg protein with a range from 4.6 × 10^10^–3.93 × 10^11^ according to the preparation (Figure 1B; Table S3). Morphological assessments revealed a spherical, bilayered, and closed membrane structure with average diameter below 100 nm according to cryo-TEM image analyses (Figure 1C). These results indicate that *L. plantarum* spontaneously releases EVs exhibiting morphology and size similar to previously described bacteria-derived EVs.

### 2.3. Lipidomic Profile of LEVs and L. plantarum

We performed lipidomic analysis for LEVs and compared the lipid profile of LEVs with that of the parent cells, *L. plantarum*. Using the multiple reaction monitoring (MRM) mode on the triple quadrupole instrument, one of mass spectrometric analyses that is highly selective and highly sensitive for targeted lipids [30], the lipid profiling was performed for glycerolipids, phospholipids, sphingolipids, and sterol lipids. Whereas glycerolipids and sterol lipids were detected as [M + NH_4_]^+^ ions, phospholipids except phosphatidylglycerol (PG), which was detected as [M + NH_4_]^+^ ions, and sphingolipids were detected as [M + H]^+^ ions (Table S1). The analyzed lipid standards showed high reproducibility and reliability by showing a coefficient of variation (CV) value under 17.6 (Table S2) [31,32]. As another quality control (QC) experiment, internal standard (IS) lipids, triacylglycerol (TG)(33:3)IS and phosphatidylcholine (PC)(20:0)IS, were analyzed twelve times to monitor lowed sensitivity during repeated MS analyses. CV values of TG(33:3)IS and PC(20:0)IS in QC experiment were 14.1 and 4.3, respectively.

**Figure 2:**
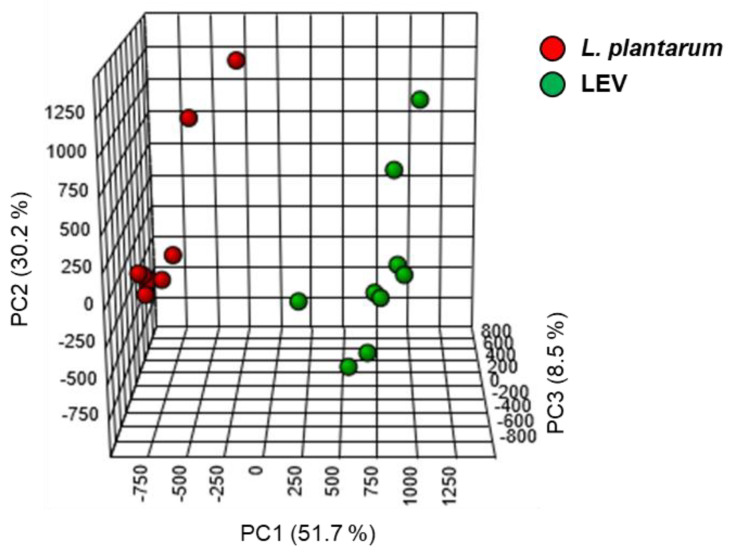
The discriminative lipidomic profile of *L. plantarum*-derived extracellular vesicles. Principal component analysis (PCA) of lipidomic information was performed based on the mass spectrometry (MS) data of LEVs and *L. plantarum*. Lipidomic data were analyzed statically using the package in MetaboAnalyst (*n* = 9 per group; three independent biological replicates and three technical replicates). X axis: principal component 1 (PC1). Y axis: principal component 2 (PC2), Z axis: principal component 3 (PC3).

**Figure 3:**
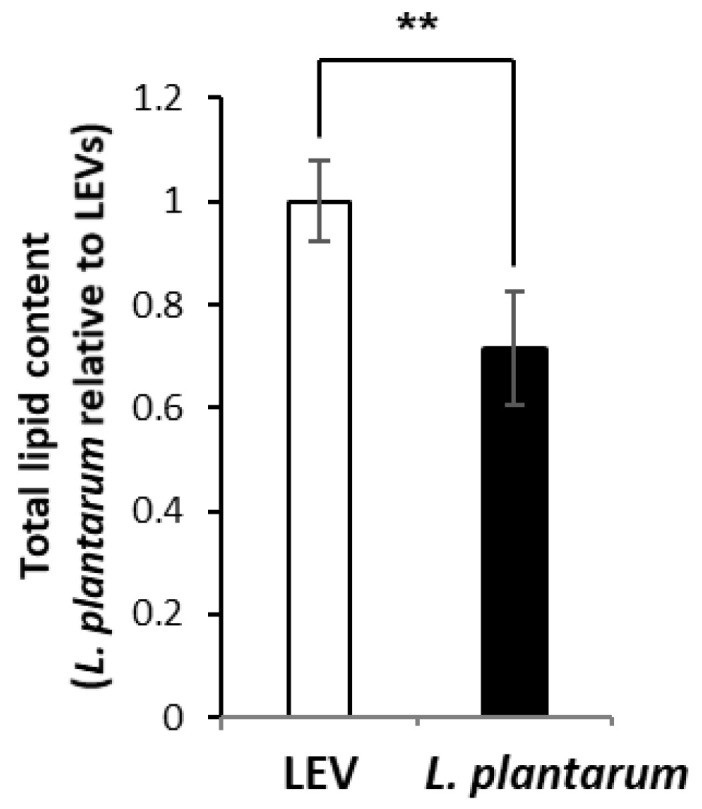
Relative expression of total lipids in LEVs and *L. plantarum*. The expression levels of total lipids were determined by summing the amounts of identified lipid species in LEVs and *L. plantarum*, respectively. The value of fold change is shown as mean ± S.D. (*n* = 9 per group; three independent biological replicates and three technical replicates). Statistical significance was analyzed by Student’s *t*-tests for two groups (** *p* < 0.01).

Based on the analysis of internal standards extracted from the sample groups, lipid extraction efficiency was no significantly different among the groups showing 69.65% ± 11.35 for medium (MRS) control, 69.60% ± 11.64 for the other strain *L. plantarum* KCTC 3108, 70.48% ± 12.17 for *L. plantarum* APsulloc 331261 (*L. plantarum*), and 66.82 ± 13.64 for LEV, but this extraction efficiency was overall lower than that from solvent control (Figure S3), possibly due to the presence of other factors including proteins and nucleic acids in addition to lipids in the sample groups. For principal component (PC) analysis of identified lipids, Z-scores for the values of PC1, PC2, and PC3 were calculated for LC-MS data obtained from nine samples each of *L. plantarum* and LEVs. The 3-3 sample for PC1, PC2 and PC3 in *L. plantarum* was slightly different from other values, but it was not significant. Similarly, the 2-3 for PC1, the 3-3 for PC2, and the 2-3 samples for PC3 in LEVs differed from the mean value, but were not significant. As a result, the lipid profiles of LEV and *L. plantarum* were distinct, showing 90.4% of variance contributed by 51.7% of PC1, 30.2% of PC2, and 8.5% of PC3 (Figure 2). A total of 320 lipid species belonging to 24 different lipid classes were quantified from both LEVs and *L. plantarum* (*n* = 9 per group; three independent biological replicates and three technical replicates) by LC-MS analysis (Table 1). Of the quantified lipids, eight species were measured only in LEVs and two in *L. plantarum* (Table S4). We determined the amounts of identified lipids in LEVs and *L. plantarum* by measuring the peak area of each lipid species using the linear calibration curves established with the internal standards representing a specific lipid family (Skyline) (Table S2; Table S5) and presented the fold changes of the quantified lipids between two groups. Overall, LEVs contain approximately a 1.4 times higher ratio of lipids/proteins than the parent cells (Figure 3). A summary of the data for fold change by lipid class in LEVs relative to *L. plantarum* is presented in Table 2. Lipid classes enriched in LEVs were diacylglycerol (DG), TG, PC, phosphatidylserine (PS) and lysophosphatidylserine (LPS), all of which were significantly increased more than two fold in LEVs. In contrast, lysophosphatidic acid (LPA) and monoacylglycerol (MG) were increased about twofold in the parental cells when compared at the same protein concentrations (Table 2). The relative abundances of 320 identified lipid species in both groups is individually displayed as a relative percentage to the highest expression value (100%) in the class (Table S6A–D). Although there was a difference in the relative abundance of lipid species within the class, the majority of lipid species were elevated in LEVs compared to *L. plantarum* except some lipid species in LPA, (lysophosphatidylglycerol) LPG, ceramide-1-phosphate (Cer1P), dihydroceramide-1-phosphate (dCer1P), sphingosine-1-phosphate (SO1P), sphinganine-1-phosphate (SA1P), and MG (Table S6A–D), which were reduced overall in LEVs.

### 2.4. Differential Enrichement of Lipid Species between LEVs and L. plantarum

**Figure 4:**
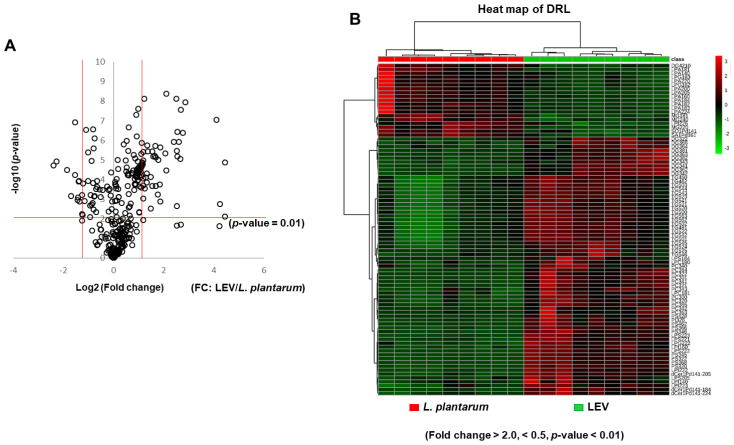
Differentially enriched lipid species in LEVs versus *L. plantarum*. (**A**) The 320 identified lipids in LEVs are listed in a volcano plot according to their statistical *p*-value and fold changes compared to those in *L. plantarum*. Red lines at left and right sides represent distinct boundaries for fold-decreased (<0.5) or -increased (>2.0) lipid species, respectively, in LEVs compared to *L. plantarum* and the horizontal red line represents a boundary for the significance (*p* < 0.01). Statistical significance was analyzed by Student’s *t*-tests for two groups. (**B**) Hierarchical clustering of differentially enriched lipid species in LEVs versus *L. plantarum* (fold change > 2.0, <0.5, *p* < 0.01). A heat map shows graphically the differential fold changes of 86 lipid species in LEVs compared to *L. plantarum*. The relative fold difference is encoded by color intensity. Red: increased; green: decreased.

We subsequently determined the fold change of individual lipid species in LEVs versus *L. plantarum* using the quantified value with a corresponding internal standard. According to a volcano plot depicting the distribution of detected lipid species by the magnitude and significance of their differential signal intensities, many lipid species were significantly fold-increased in LEVs compared to *L. plantarum* (Figure 4A), which is consistent with the result shown in fold change of lipid classes (Table 2). In the plot, the vertical red lines represent distinct boundaries showing at least twofold difference between LEVs and *L. plantarum* for increased or decreased lipids, respectively, and the horizontal red line represents a boundary for the significance (*p* = 0.01) (Figure 4A). Of the 320 identified lipids whose relative enrichment between two groups is shown in a heat map (Figure S4), 86 lipid species (26.9%) showed a statistically significant difference between the groups with 67 (77.9%) showing increase (fold change > 2; *p* < 0.01) and 19 (22.1%) showing decrease (fold change < 0.5; *p* < 0.01) in LEVs compared to *L. plantarum* (Figure 4A). These significantly and differentially fold-changed lipid species are displayed on a heat map (Figure 4B) and the list are described in Table 3 and Table 4, respectively, together with the quantitative values of differentially increased or decreased lipid species (Tables S7 and S8). The largest number of increased lipid species in LEVs is taken up by TG (21) followed by PC (12), DG (10), PS (8), LPS (5), LPI (4), dCer1P (3), LPC (2), LPG (1), and PI (1) (Table 3). More than half (10) of the decreased lipid species in LEVs are LPA (Table 4). Compared to *L. plantarum*, LPS(18:4) and PC(32:2) were markedly increased in LEVs, showing the highest enrichment of greater than 21-fold, and three PC species including PC(32:2), PC(34:2), and PC(30:1) were ranked in the top five (Table 3). In addition, among the eight lipid species detected only in LEVs, seven were phospholipids (Table S4). These results suggest that LEVs are enriched with phospholipids including PC species in a high rank and these could be potential characteristics of LEVs.

### 2.5. Comparison of the Lipid Composition in LEVs Versus L. plantarum

**Figure 5:**
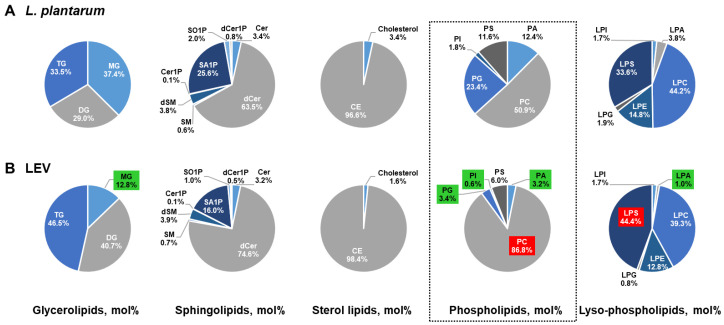
Lipid composition and proportion in *L. plantarum* versus LEVs. In pie diagrams for *L. plantarum* (**A**) and LEVs (**B**), the composition by lipid class (glycerolipids, sphingolipids, sterol lipids, phospholipids, and lyso-phospholipids) is expressed as a percentage of lipid category (mol% of lipid category) which was defined as the sum of the amount of identified lipid species (nmol/mg proteins). The markedly changed lipid classes in LEVs compared to *L. plantarum* are designated by red (increased) or green (decreased).

We further examined whether there are compositional differences among lipid classes in LEVs and *L. plantarum* by accessing the proportion of each lipid class in lipid category (mol% of lipid category). LEVs showed a relatively high ratio in TG (46.5%) and DG (40.7%) but low in MG (12.8%), while *L. plantarum* showed similar ratios in composition (Figure 5A,B). Neither group showed considerable difference in the proportion of sphingolipids or sterol lipids (Figure 5A,B). However, as expected from the differentially increased lipid species in LEVs (Table 3), there was notable difference in the composition and proportion of phospholipid class. Among 11 phospholipid classes, the proportion of PC and LPS was increased in LEVs compared to in the parent cells, while that of PA, PG, PI, PS, LPA, LPC, LPE, LPG, and LPI was decreased (Figure 5A,B). Especially, the portion of PC increased 35.9% in LEVs, accounting for 86.8% in LEVs and 50.9 % in *L. plantarum*, and the portion of PA, PG, PI, and LPA, decreased more than threefold in LEVs. These results suggest that there is a difference in the composition and proportion of lipid classes, especially phospholipids, between LEVs and their parent cells, which may reflect the compositional and structural characteristics of EVs, lipid bilayer-enclosed vesicles.

## 3. Discussion

In this study, we showed significant and quantitative differences in the lipid composition of LEVs from that of the parent *L. plantarum* using lipidomic LC-MS analysis, and finally verified that, in comparison to the parent cells, 67 and 19 out of 320 identified lipid species were significantly increased and decreased in LEVs, respectively. Given that most EVs derived from microorganisms and mammalian cells have been analyzed in their proteomes but only a handful of studies report a lipidomics analysis of EVs derived from either mammalian cells or body fluids (urine and plasma) of healthy persons or patients [33,34,35,36,37,38,39,40]; this is, to our knowledge, the first study reporting the lipidomic analysis of probiotic EVs.

When performing the LC-MS analysis repeatedly for the technical replicates, it shows a problem of gradually lower sensitivity. Therefore, it is important to minimize the variation that appears during repeated MS analyses (between technical replicates). We confirmed the sensitivity of LC-MS through QC analysis and obtained a stable CV value under 20. Although there were some differences in sample variation in PCA analysis and the heat map, which are possibly due to lower sensitivity during repeated MS analyses for technical replicates or different culture conditions and EV purification procedures for biological replicates, it seemed that there was no problem in comparing lipid expression patterns in LEV and the parent cells according to stable CV values of IS lipids.

In LEVs, phospholipids, including PC in particular, were elevated compared to the parent cells in agreement with EVs being lipid bilayer-enclosed structures where phospholipids are a major component because of their amphiphilic characteristic and provide EVs with stability and structural rigidity together with sphingolipids. It was believed that many prokaryotes lack PC. However, there was the first report to claim that PC was detected in *Lactobacillus casei* using thin-layer chromatography [41]. More recently, PC has been found in significant amounts in membranes of diverse Gram-negative bacteria living in association with eukaryotes for nitrogen fixation (mainly Rhizobiales and Rhodobacterales) and in those of some Gram-positive bacteria [42,43,44]. Of Gram-positive bacteria, Actinobacteria isolated from reed or soil contain PC as one of the major polar lipids [45,46]. In Gram-positive *Lactobacillus* species, based on lipidomic profiling on *L. acidophilus* and *L. gasseri*, some lipid species of PC and LPC were significantly elevated in *L. gasseri* compared to *L. acidophilus* (Supplementary data) [47]. Based on genomic sequence data looking for the presence of either the phospholipid N-methyltransferases (PMT) or the phosphatidylcholine synthase (PCS) pathways, which are two of four different pathways for PC synthesis, about 15% of bacteria could potentially form PC [42,43,44]. Given that different bacterial species display different membrane compositions and even membrane composition of a single species is not consistent according to the environmental conditions that bacteria are exposed to [43,48], the bacterial strain *L. plantarum* APsulloc isolated from green tea leaves and grown under specific media and culture conditions may potentially release PC-enriched, beneficial EVs in a relatively high amount compared to other Gram-positive bacteria. In the sense that PC is a major phospholipid of mammalian membrane, we speculate that the elevated PC contents would be helpful to improve the attachment and association of LEVs to the human cells, thereby efficiently delivering the probiotic properties toward the human cells. Especially, PC(32:2), PC(34:2), and PC(30:1) showed, respectively, over 21-, 18-, and 17-fold enhancement difference between LEVs and the parent cells and positioned in the top five among differentially increased lipid species (Table 3). Therefore, these species, together with LPS(18:4), the most increased species in LEVs, could be used as lipid biomarkers to determine the presence of LEVs in the materials containing LEVs developed for clinical or cosmeceutical applications. In this respect, of eight lipid species detected only in LEVs, PE(30:0), DG(42:8), and PE(32:3) were detectable in considerable amounts, suggesting potent alternative indicators for LEVs.

To clarify that there may be basal lipid signals due to the culture medium components, we included medium control for the analysis and recognized that the peak intensity related to target lipid species was weak but detectable in medium control (Figure S3B), suggesting that there were some of basal lipidomic signals from the medium components. However, the analyzed lipid classes (TG, Cer, SM, PC, LPC, and CE/Chol) were clearly fold-increased in LEVs compared to medium control and *L. plantarum* APsulloc 331261 where the increase was not as clear as that in LEVs but still increased compared to medium control (Figure S3B). Interestingly, lipid classes including Cer, SM, PC, and LPC were overall increased in *L. plantarum* APsulloc 331261 compared to the other strain KCTC 3108, due to related lipid species increased more than twofold in *L. plantarum* APsulloc 331261 (Figure S3C,D). These results suggested that lipid classes such as Cer, SM, CE/Chol, and in particular, PC, which all are expected to be less frequent in prokaryotes, could be present in *L. plantarum* APsulloc 331261-derived EVs in relatively high levels and this lipidomic profile may affect the functional role mediated by LEVs in the communication between bacteria and human cells.

Besides PC, other lipids including TG, PI, sphingolipids, and sterol lipids are less frequent in prokaryotes [43,49,50]. Contrary to mammalian cells where cholesterol and sphingolipids including ceramide and sphingomyelin are ubiquitous components of the cytoplasmic membrane and have functions in the formation of lipid rafts, thereby of EVs [20,51], sphingolipids occur only in a few bacteria and not much is known about their biosynthesis and functions in bacteria [43,52]. Based on the analysis of lipid composition of two bacterial EVs derived from *L. plantarum* APsulloc and *P. acnes* [53], respectively, the compositions of sphingolipids were below 10% in total lipids (Figure S5), suggesting that low sphingolipid composition may be one big difference in bacterial EVs as compared to mammalian EVs. Given that sphingolipids including Cer participate in vesicle formation and secretion by increasing membrane fluidity and are important regulators for bacterial entry and adherence [54], bacterial EVs possessing different compositions and proportions in sphingolipids according to species may have different potential to affect host cells. Considering the technical advances in lipidomic analysis [30,55], we believe that diverse lipid species will be increasingly detected in bacterial membranes and their extracellular vesicles, although these comprise a minority of the total lipid signal. It is also known that there is a plasticity in the membrane lipid composition of several bacteria under different growth conditions, which was exemplified by CE formation in several members of *Actinobacteria* and *Proteobacteria* and cardiolipin and ornithine lipids synthesis in *Streptomyces* [48,50]. In addition, phosphatidic acid, the key precursor to all phospholipid species in bacteria, can be either synthesized via *de novo* type II fatty acid synthesis system (FASII) or from exogenous fatty acids which are converted into acyl-phosphates or become acyl-acyl carrier protein [56,57,58]. In the most extreme example, it is known that certain *Lactobacillus* species don’t encode the genes for FASII and synthesize phospholipids entirely from exogenous fatty acids [58], suggesting that the culture conditions or habit environments for *Lactobacillus* species, for example, *L. plantarum* APsulloc 331261 could have directly influence on their membrane lipid compositions. Therefore, it would be worthy to examine whether the lipid composition of *L. plantarum* and its EVs is changed according to the medium constituents (MRS vs. a specialized medium) and culture conditions (aerobic vs anaerobic).

Interestingly, derivatives of phospholipids generated by autotaxin (a lysophospholipase D), LPC and LPA in particular, were diminished in LEVs compared to corresponding parent cells (Figure 5). In mammalian cells, LPA is a signaling molecule stimulating cell survival, proliferation, and migration via its G-protein-coupled receptors, by which it can increase angiogenesis and metastasis in cancer and drive chronic inflammatory conditions [59,60]. Thus, aberrant LPA-signaling networks are likely to be related to human diseases including cancer or inflammatory disorders and may be suitable targets for clinical application [60]. In particular, bacteria such as *Legionella pneumophila* are known to utilize phospholipase A_2_ end-products (fatty acids and lysophospholipids) to cause host cell (macrophage) apoptosis through cytochrome C release from mitochondrial membranes [61]. This suggests that bacterial lysophospholipids such as LPA and its precursor LPC could disturb the signaling networks in host cells; therefore, *L. plantarum*-derived EVs possessing less LPA might be less toxic (safer) than the parent cells for humans, which is important when considered as a probiotic material. On the other hand, *P. acnes*-derived EVs might be more toxic to humans due to an enriched LPA compared to its parent cells [53], suggesting that distinctive composition of phospholipids among bacteria-derived EVs could be one mechanism for their differential pathogenicity toward host cells. With regard to this speculation, it would be worthy to compare the lipidomic and compositional analyses of *L. plantarum* and *P. acnes* and their EVs with those of *S. aureus* and SEVs, which are representative pathogenic bacteria and derivatives related to the occurrence and pathogenesis of atopic dermatitis [22,62].

## 4. Materials and Methods

### 4.1. Materials

HPLC-grade methanol, acetonitrile, water, chloroform, and isopropyl alcohol were purchased from J.T. Baker (Phillipsburg, NJ, USA). HPLC-grade formic acid, hydrochloric acid, ammonium formate, and acetic acid were purchased from Sigma-Aldrich (St. Louis, MO, USA). Lipid standards used in this study were purchased from Larodan Fine Chemicals AB [Cholesteryl ester; CE (10:0), Monoacylglycerols; MG (15:1), Diacylglycerols; DG (8:0-8:0), Triacylglycerols; TG (11:1-11:1-11:1)] and Avanti Polar Lipids, Inc. [Phosphatidylcholine; PC (10:1-10:1), Lysophosphatidylcholine; LPC (13:0), Phosphatidylethanolamine; PE (10:1-10:1), Lysophosphatidylethanolamine; LPE (14:0), Phosphatidylglycerol; PG (10:1-10:1), Lysophosphatidylglycerol; LPG (14:0), Phosphatidic acid; PA (10:1-10:1), Lysophosphatidic acid; LPA (17:0), Phosphatidylserine; PS (10:1-10:1), Lysophosphatidylserine; LPS (17:1), Phosphatidylinositol; PI (16:0), Lysophosphatidylinositol; LPI (13:0), Ceramide; Cer (d18:1-12:0), Dihydroceramide; dCer (d18:0-12:0), Ceramide-1-phosphate; Cer1P (d18:1-12:0), Dihydroceramide-1-phosphate; dCer1P (d18:0-16:0), Sphingosine; SO (17:1), Sphinganine; SA (17:0), Sphingosine-1-phosphate; SO1P (17:1), Sphinganine-1-phosphate; SA1P (17:0), Sphingomyelin; SM (d18:1-12:0), Dihydrosphingomyelin; dSM (d18:1-12:0)].

### 4.2. Isolation and Identification of L. plantarum APsulloc

Green tea leaves were harvested from Dosun green tea garden in Jeju, South Korea. Two hundred grams of green tea leaves were washed twice with distilled water and mixed with salt equivalent to 8% (*w/w*) of green tea leaves weight, and left at room temperature for 3 h. The salted green tea leaves are mixed with 1000 mL of a 1% fructooligosaccharide solution and incubated for three days at 32 °C. The pH change in the solution was monitored and when the solution showed acidic pH (below pH 5.0), it was inoculated on to a de Man, Rogosa and Sharpe (MRS) agar plate (Lactobacilli MRS agar; BD Difco, Franklin Lakes, NJ, USA) and incubated for two days at 32 °C in an anaerobic chamber (Bactron; Sheldon Manufacturing, Cornelius, OR, USA) connected with a gas cylinder containing 5% H_2_, 5% CO_2_, and 90% N_2_ for putative LAB growth. After monitoring the morphology of colonies, putative LAB colonies were picked and reinoculated onto a MRS agar plate, and grown in MRS broth (Lactobacilli MRS broth; BD Difco, Franklin Lakes, NJ, USA) at 32 °C under anaerobic conditions. To identify the obtained isolates, fifteen colonies were selected for partial 16S rDNA sequencing. Chromosomal DNA was purified using Wizard genomic DNA purification kit (Promega, Madison, WI). PCR were performed on a thermal cycler using universal primers 27F (5′-AGAGTTTGATCATGGCTCAG-3′) and 1492R (5′-GGATACCTTGTTACGACTT-3′) [63]. The amplified PCR products (~ 1 kb) were purified using Wizard SV gel and PCR clean-up system (Promega) and sequenced using ABI PRISM 3700 DNA analyzer (Waltham, MA, USA). The partial 16S gene sequences were compared with sequences on the nucleotide data base of GenBank (NCBI) using BLASTN and the similarity between sequences was analyzed by the Clustal W and Mega 7 programs [64]. The phylogenic relationship of LAB colony (bacterial strain APsulloc 331261) with *L. plantarum* was verified through the analysis of neighbor-joining tree using partial 16S rDNA sequencing.

### 4.3. Isolation of EVs from the Bacterial Culture Medium

*Lactobacillus plantarum* APsulloc 331261 (deposit number: KCCM11179P) was grown in MRS broth for 24 h at 37 °C under anaerobic conditions, and was then subcultured under the same condition following 1 to 100 dilution. When the culture reached an optical density of 1.0–1.5 at 600 nm (O.D._600_), cells (≥ 2.7 × 10^9^ colony-forming units (cfu)/mL) were pelleted by sequential centrifugation at 3000× *g* for 10 min at 4 °C and 10,000× *g* for 20 min at 4 °C and frozen for lipid extraction. *Lactobacillus plantarum* APsulloc 331261-derived EVs (LEVs) were purified from culture supernatant according to purification methods for Gram-positive bacterial EVs [17]. In brief, the supernatants were filtered using a 0.45-μm vacuum filter (Millipore, Billerica, MA, USA) and then subjected to ultracentrifugation at 150,000× *g* for 3 h at 4 °C (Type 45 Ti rotor, Beckman Coulter, Brea, CA, USA). After centrifugation, the pellet was diluted in HEPES-buffered saline (HBS), and the total protein concentration was determined using a Bradford assay (Bio-Rad Laboratories, Hercules, CA, USA). For lipidomic analysis, LEVs were further purified by OptiPrep density gradient (Sigma-Aldrich, St. Louis, MO, USA) according to the manufacturer’s instructions and a previously described method [29] with some modifications. OptiPrep^TM^ (60 *w*/*v*% iodixanol in distilled water) was diluted to 50%, 35%, and 20% in 0.25 M sucrose buffer (0.25 M sucrose, 150 mM NaCl, 20 mM HEPES pH 7.4). A bottom-loading and discontinuous gradient was formed by layering 2.5 mL of each solution in 10 polypropylene centrifugation tubes (Beckman Coulter, Brea, CA, USA) and centrifuged at 200,000× *g* at 4 °C for 2 h (SW 41 Ti rotor, Beckman Coulter). The EV fraction at density between 1.17~1.24 was collected using a 1 mL-syringe. To remove iodixanol, the fraction was diluted in 60 mL of HBS, ultracentrifuged at 150,000× *g* at 4 °C for 3 h, and then resuspended with 200 μL of HBS.

### 4.4. Analysis of LEVs

The purified LEV size was measured by dynamic light scattering using a Zetasizer Nano ZS (Malvern Instruments, Worcestershire, UK) and analyzed using Dynamic V6 software. The particle numbers of purified LEVs were determined by tunable resistive pulse sensing using a qNano-Gold (IZON Science LTD, Christchurch, New Zealand). Cryo-TEM analysis of LEVs was performed as described previously [21]. Briefly, 3 µL of LEVs were placed onto both sides of a Quantifoil TEM grid with a hole diameter of 1.2 μm and inter-hole distance of 1.3 μm. The TEM grid was blotted for 1.5 s and plunged into liquid ethane using a Cryoplunge 2 system (Gatan Inc., Pleasanton, CA, USA). Prepared TEM samples were stored in liquid nitrogen before TEM observation. Samples were examined under an FEI Tecnai F20 electron microscope (Hillsboro, OR, USA) operating at 120 kV.

### 4.5. Lipid Extraction

Each lipid standard was dissolved in methanol or chloroform and diluted to 100 ng/μL for extraction. A two-step extraction method was performed for high efficiency extraction of polar and nonpolar lipids [65]. Medium control (MRS), *L. plantarum* KCTC 3108 and *L. plantarum* APsulloc 331261 which were washed three times with phosphate buffered saline (PBS) to remove media residue, and LEVs were treated or lysed by adding 5 μL of 0.1% sodium dodecyl sulfate (SDS) in PBS into 45 μL of each sample in PBS (final 0.01% SDS in PBS) and incubating for 20 min at 37 °C. After sonication (program: pulse on for 5 s, pulse off for 10 s, 10% amplitude) for 3 min on ice using a probe sonicator (Branson, Danbury, CT, USA), the lysed samples were centrifuged at 13,800× *g*, 20 min at 4 °C. The supernatants were collected and protein concentration was determined using a Pierce bicinchoninic acid (BCA) protein assay kit (Thermo Fisher Scientific, Waltham, MA, USA). Samples set to contain the same amount of 30 μg protein in 50 μL of 0.01% SDS in PBS were added to methanol/chloroform (2:1, *v*/*v*; 660 μL:330 μL) with 20 μL of the above-mentioned solution as internal standard (IS) lipids (a mixture of IS lipids). Samples were subsequently vortexed 3 × 30 s and incubated for 10 min at room temperature. After centrifugation (13,800× *g*, 2 min at 4 °C), the supernatants were transferred to new 1.5 mL tubes. The remaining pellets were dissolved in chloroform/methanol/37% HCl (40:80:1, *v*/*v*/*v*) and incubated for 15 min at room temperature with vortexing three times for 30 s. Then, 250 μL of chloroform and 450 μL 0.1 M HCl were added to samples. Next, the samples were vortexed vigorously for 1 min and centrifuged at 6500× *g* for 2 min at 4 °C. The bottom of the organic phase was collected and pooled with that extracted before. Subsequently, all samples were split in half and dried by Speed Vac concentrator. One of the dried sample pairs was then dissolved in 100 μL of solvent A/solvent B (2:1, *v*/*v*) for neutral and positive lipid analysis, and the other half of the pair was dissolved in 100 μL of methanol for trimethylsilyldiazomethane (TMSD) methylation [66] to analyze anionic lipids.

### 4.6. Global Lipid Analysis Using LC-MS

The HPLC analysis was performed on Waters Acquity UPLC instrument (Waters, Milford, MA, USA). A Hypersil Gold column (2.1 × 100 mm ID; 1.9 μm, Thermo Fisher Scientific, Waltham, MA, USA) was used for separation of lipids. Solvent A consisted of methanol-acetonitrile-water (19:19:2, *v*/*v*) with 20 mM ammonium formate and 0.1% (*v*/*v*) formic acid and solvent B consisted of isopropyl alcohol with 20 mM ammonium formate and 0.1% (*v*/*v*) formic acid. The flow rate was 0.3 mL/min and the injection volume was 4 μL for each run. A 33 min gradient was performed as follows: 0–5 min, B 5%; 5–15 min, B 30%; 15–22 min, B 90%, and maintained for 5 min. Finally, the column equilibrated at B 5% for 5 min before subsequent analysis. All lipid analysis of samples was performed by QTRAP 5500 (AB Sciex) hybrid, triple quadrupole, linear ion trap mass spectrometer equipped with a Turbo V ion source, together with the Analyst 1.5.1 software package (AB Sciex, Foster City, CA, USA). Ultra-high-purity nitrogen gas was used for the collision. The parameters of operating source conditions were as follows: capillary voltage 5500 V; nebulizer gas (GS1) = 30–50 psi, heater gas (GS2) = 30–50 psi, collision gas setting (CAD) = high, source temperature (Temp) = 400–500 °C. All lipids were analyzed using optimized conditions and achieved in the multiple reaction monitoring (MRM) mode using computed transitions for each lipid class. For the MRM scan mode, the parameters are follows: different MRM transitions, run time, 22 min, entrance-potential, 10; collision cell exit potential (CXP), 15. The information for precursor and product ions per lipid target, the MS/MS collision energy, and the declustering potential, which was optimized for each lipid transition, is presented in Table S1. LC-MS analysis for *L. plantarum* APsulloc 331261 and LEV was repeated nine times, respectively (*n* = 9 per group; three independent biological replicates and three technical replicates).

### 4.7. Processing of Individual Data Obtained by MRM

The MS data files were processed using the Analyst 1.5.1 software package (AB Sciex, Foster City, CA, USA). Targeted lipids on the MRM list, including precursor and product ions, were assigned by comparisons of retention times with internal standards. Skyline software package (MacCoss Laboratory, Seattle, WA, USA) was used with an in-house database to determine the peak area of each assigned lipid and all data were normalized by respective internal standard. The retention times, linear ranges, and limits of detection (LOD) for internal standards were summarized in Table S2. Technical outliers and lipid species were removed based on LOD of the analyzed standard lipids. Retention time (RT) values that appear differently depending on the length of fatty acids of lipid species were assigned by referring to LIPID MAPS^®^ Lipidomics Gateway, https://www.lipidmaps.org/. The concentrations of lipids are presented as nmol/mg protein or as mol% of total lipids or lipid category.

Quality control (QC) experiments were performed to monitor the overall quality of the lipid extraction and MS analyses [31,32]. In addition to monitoring a coefficient of variation (CV) value for each IS lipid in all analyzed samples (Table S2), a total of twelve MS analyses were performed for two IS lipids, TG (33: 3)IS and PC (20: 0)IS to monitor lowed sensitivity during repeated MS analyses. The efficiency of lipid extraction was calculated based on the MRM analyses for 14 IS lipids [DG(16:0)IS, TG(33:3)IS, PC(20:0)IS, LPC(13:0)IS, PE(20:0)IS, LPE(14:0)IS, SO1P(d17:1)IS, Cer(d18:1–12:0)IS, SM(d18:1–12:0)IS, SA1P(d17:0)IS, dCer(d18:0–12:0)IS, dSM(d18:1–12:0)IS, PG(20:0)IS, and LPG(14:0)IS] after their extraction from the samples of medium control (MRS), *L. plantarum* KCTC 3108, *L. plantarum* APsulloc 331261, and LEVs at the same protein amount (30 μg), as described in Section 2.5. The extraction efficiency of each IS lipid in the sample groups was calculated relatively from measured MRM value of that in IS alone in solvent (methanol), and the lipid extraction efficiency of samples was, therefore, expressed as the average value of the extraction efficiency of each IS lipid. Hierarchical clustering of data was performed using the MetaboAnalyst website [67].

## 5. Conclusions

In conclusion, our study using quantitative and comparative LC-MS analysis demonstrated that the lipidomic profile of LEVs is different from the parent cells, showing significant increase in 67 lipid species but decrease in 19 species compared to *L. plantarum*. In addition, there seems to be a notable difference in the composition and proportion of phospholipids between EVs and the parent cells. Given that lipids are important components of bioactive vesicles, EVs, quantitative and comparative analyses of EV lipids may improve our understanding of vesicle biogenesis and lipid-mediated intercellular communication within or between living organisms.

## Figures and Tables

**Table 1:** The detected lipid classes by LC-MS from both LEVs and *L. plantarum*. The number of identified lipid species in each lipid class was shown in descending order.

Lipid Class	Lipid Species Quantitated
Phosphatidic acid (**PA**)	**50**
Triacylglycerol (**TG**)	**43**
Diacylglycerol (**DG**)	**23**
Phosphatidylserine (**PS**)	**23**
Phosphatidylcholine (**PC**)	**20**
Monoacylglycerol (**MG**)	**17**
Lysophosphatidic acid (**LPA**)	**17**
Cholesterylester (**CE**)	**16**
Lysophosphatidylserine (**LPS**)	**16**
Lysophosphatidylglycerol (**LPG**)	**13**
Dihydroceramide (**dCer**)	**11**
Phosphatidylglycerol (**PG**)	**11**
Lysophosphatidylinocitol (**LPI**)	**11**
Lysophosphatidylcholine (**LPC**)	**8**
Ceramide (**Cer**)	**7**
Phosphatidylinositol (**PI**)	**7**
Dihydroceramide-1-phosphate (**dCer1P**)	**6**
Ceramide-1-phosphate (**Cer1P**)	**5**
Dihydrosphingomyelin (**DSM**)	**4**
Sphingomyeline (**SM**)	**3**
Sphinganine-1-phosphate (**SA1P**)	**3**
Sphingosine-1-phosphate (**SO1P**)	**3**
Lysophosphatidylethanolamine (**LPE**)	**2**
Cholesterol	**1**
**Total**	**320**

**Table 2:** Summary for the fold change of lipid classes in LEVs relative to *L. plantarum*. The value of fold change is shown as mean ± S.D. (*n* = 9 per group; three independent biological replicates and three technical replicates). Statistical significance was analyzed by Student’s *t*-tests for two groups.

Lipid Category	Lipid Class	Fold Change ^a^ (Mean ± S.D.)	*p*-Value
**Glycerolipids**	**MG**	0.505 ± 0.0898	4 × 10^−9^
	**DG**	2.04 ± 0.476	1 × 10^−4^
	**TG**	2.26 ± 0.625	1 × 10^−5^
**Sphingolipids**	**Cer**	1.039 ± 0.0627	3 × 10^−1^
	**dCer**	1.36 ± 0.188	2 × 10^−2^
	**SM**	1.194 ± 0.148	7 × 10^−3^
	**dSM**	1.15 ± 0.062	2 × 10^−2^
	**Cer1P**	0.91 ± 0.0496	6 × 10^−2^
	**SA1P**	0.685 ± 0.0877	1 × 10^−3^
	**SO1P**	0.536 ± 0.0824	3 × 10^−6^
	**dCer1P**	0.682 ± 0.285	2 × 10^−1^
**Glycero-phospholipids**	**PA**	1.09 ± 0.0318	5 × 10^−4^
	**PC**	6.92 ± 1.44	3 × 10^−4^
	**PG**	0.573 ± 0.282	8 × 10^−2^
	**LPA**	0.491 ± 0.0811	6 × 10^−4^
	**LPC**	1.63 ± 0.215	9 × 10^−3^
	**LPE**	2.41 ± 2.696	2 × 10^−1^
	**LPG**	0.812 ± 0.215	1 × 10^−1^
	**PI**	1.52 ± 0.122	6 × 10^−6^
	**LPI**	1.77 ± 0.167	3 × 10^−4^
	**PS**	2.22 ± 0.204	8 × 10^−7^
	**LPS**	2.23 ± 0.763	2 × 10^−3^
**Sterol lipids**	**Cholesterol**	0.553 ± 0.193	2 × 10^−3^
	**CE**	1.26 ± 0.134	9 × 10^−2^

**^a^**, LEV/*L. plantarum*.

**Table 3:** **The list of differentially increased lipid species in LEVs compared to *L. plantarum***. The data are shown as mean ± S.D. of the fold increase with *p*-value (*n* = 9 per group; three independent biological replicates and three technical replicates). The list is displayed in descending order of fold change. Statistical significance was analyzed by Student’s *t*-tests for two groups.

No.	Lipid	Fold Change ^a^ (Mean ± S.D.)	*p*-Value
1	**LPS(18:4)**	21.66 ± 13.15	8 × 10^−3^
2	**PC(32:2)**	21.64 ± 3.35	1 × 10^−5^
3	**PC(34:2)**	18.84 ± 4.08	2 × 10^−3^
4	**PC(30:1)**	17.21 ± 1.35	9 × 10^−8^
5	**PS(32:2)**	7.32 ± 0.94	1 × 10^−8^
6	**LPI(22:2)**	6.79 ± 0.54	4 × 10^−7^
7	**LPS(22:2)**	6.43 ± 0.81	3 × 10^−8^
8	**PC(34:1)**	6.29 ± 0.96	9 × 10^−6^
9	**DG(36:2)**	6.04 ± 1.13	1 × 10^−6^
10	**PC(32:1)**	5.85 ± 0.5	6 × 10^−6^
11	**LPS(22:3)**	5.84 ± 0.84	4 × 10^−7^
12	**PC(36:3)**	5.75 ± 1.33	2 × 10^−3^
13	**dCer1P(d14:1–18:4)**	5.74 ± 0.75	3 × 10^−6^
14	**PS(36:8)**	5.69 ± 0.66	8 × 10^−9^
15	**dCer1P(d14:1–20:5)**	5.26 ± 1.86	1 × 10^−6^
16	**DG(36:3)**	4.3 ± 1.2	3 × 10^−5^
17	**PS(34:5)**	4.3 ± 0.65	4 × 10^−9^
18	**DG(34:1)**	4.17 ± 0.81	5 × 10^−6^
19	**TG(52:5)**	3.64 ± 1.21	1 × 10^−3^
20	**LPS(22:1)**	3.62 ± 0.36	2 × 10^−6^
21	**dCer1P(d14:1–22:4)**	3.6 ± 3.31	2 × 10^−4^
22	**DG(34:2)**	3.46 ± 0.71	1 × 10^−5^
23	**LPC(18:1)**	3.41 ± 2.7	6 × 10^−4^
24	**LPC(18:0)**	3.23 ± 0.88	2 × 10^−4^
25	**LPG(22:2)**	3.2 ± 0.45	7 × 10^−6^
26	**PC(38:4)**	3.18 ± 0.5	2 × 10^−6^
27	**PC(30:0)**	3.1 ± 0.42	6 × 10^−6^
28	**PI(32:6)**	2.97 ± 0.55	6 × 10^−6^
29	**DG(38:1)**	2.91 ± 0.54	2 × 10^−4^
30	**PC(32:0)**	2.89 ± 0.29	4 × 10^−6^
31	**TG(54:6)**	2.8 ± 0.63	3 × 10^−3^
32	**DG(34:3)**	2.78 ± 0.37	7 × 10^−5^
33	**PC(38:5)**	2.58 ± 0.61	4 × 10^−6^
34	**LPI(16:0)**	2.55 ± 0.23	2 × 10^−6^
35	**DG(38:2)**	2.51 ± 0.57	1 × 10^−4^
36	**TG(54:5)**	2.5 ± 0.28	5 × 10^−5^
37	**PS(32:0)**	2.44 ± 0.18	1 × 10^−9^
38	**TG(52:4)**	2.44 ± 0.63	8 × 10^−5^
39	**LPI(22:3)**	2.37 ± 0.94	3 × 10^−4^
40	**PS(36:6)**	2.32 ± 0.13	7 × 10^−9^
41	**PS(42:8)**	2.28 ± 0.68	2 × 10^−4^
42	**TG(56:3)**	2.27 ± 0.46	4 × 10^−6^
43	**LPS(20:5)**	2.26 ± 0.45	9 × 10^−4^
44	**DG(36:6)**	2.26 ± 0.35	9 × 10^−5^
45	**TG(54:3)**	2.26 ± 0.62	1 × 10^−5^
46	**TG(52:1)**	2.23 ± 0.53	2 × 10^−5^
47	**TG(52:3)**	2.2 ± 0.66	1 × 10^−5^
48	**PS(46:2)**	2.2 ± 0.53	1 × 10^−5^
49	**PS(34:6)**	2.18 ± 0.28	1 × 10^−6^
50	**TG(52:0)**	2.18 ± 0.61	2 × 10^−5^
51	**TG(54:4)**	2.15 ± 0.61	2 × 10^−5^
52	**TG(56:2)**	2.15 ± 0.72	5 × 10^−6^
53	**DG(32:2)**	2.14 ± 0.42	2 × 10^−4^
54	**TG(54:2)**	2.14 ± 0.74	2 × 10^−5^
55	**PC(34:3)**	2.13 ± 0.31	4 × 10^−5^
56	**TG(50:3)**	2.12 ± 0.62	2 × 10^−5^
57	**TG(54:1)**	2.11 ± 0.58	6 × 10^−5^
58	**TG(52:2)**	2.11 ± 0.84	3 × 10^−5^
59	**TG(50:2)**	2.07 ± 0.66	2 × 10^−5^
60	**TG(50:0)**	2.07 ± 0.82	8 × 10^−5^
61	**DG(36:4)**	2.07 ± 0.4	2 × 10^−4^
62	**TG(50:1)**	2.06 ± 0.65	3 × 10^−5^
63	**TG(48:1)**	2.05 ± 0.6	3 × 10^−5^
64	**LPI(14:0)**	2.03 ± 0.44	5 × 10^−3^
65	**PC(34:0)**	2.02 ± 0.33	5 × 10^−3^
66	**TG(46:0)**	2 ± 0.6	3 × 10^−5^
67	**TG(48:2)**	2 ± 0.53	2 × 10^−5^

Differentially increased lipid list (Fold change > 2.0, *p*-value < 0.01); **^a^**, LEV/*L. plantarum*.

**Table 4:** The list of differentially decreased lipid species in LEVs compared to *L. plantarum*. The data are shown as mean ± S.D. of the fold-decrease with *p*-value (*n* = 9 per group; three independent biological replicates and three technical replicates). The list is displayed in descending order of fold change. Statistical significance was analyzed by Student’s *t*-tests for two groups.

No.	Lipid	Fold Change ^a^ (Mean ± S.D.)	*p*-Value
1	**SO1P(d14:1)**	0.49 ± 0.05	4 × 10^−6^
2	**DG(42:10)**	0.49 ± 0.05	1 × 10^−4^
3	**MG(18:1)**	0.46 ± 0.02	3 × 10^−7^
4	**LPA(18:3)**	0.44 ± 0.09	1 × 10^−3^
5	**LPI(22:5)**	0.43 ± 0.11	2 × 10^−4^
6	**LPG(18:3)**	0.42 ± 0.11	6 × 10^−3^
7	**LPA(22:4)**	0.42 ± 0.15	6 × 10^−4^
8	**LPA(14:0)**	0.41 ± 0.08	6 × 10^−3^
9	**LPA(22:0)**	0.39 ± 0.08	1 × 10^−3^
10	**LPA(20:0)**	0.38 ± 0.05	1 × 10^−4^
11	**LPA(18:2)**	0.38 ± 0.06	1 × 10^−4^
12	**LPA(16:0)**	0.37 ± 0.07	8 × 10^−4^
13	**LPG(18:2)**	0.36 ± 0.09	1 × 10^−3^
14	**SA1P(d16:1)**	0.34 ± 0.05	1 × 10^−7^
15	**LPA(20:5)**	0.34 ± 0.05	6 × 10^−5^
16	**LPA(18:4)**	0.3 ± 0.06	7 × 10^−4^
17	**MG(24:0)**	0.27 ± 0.07	3 × 10^−5^
18	**MG(22:0)**	0.2 ± 0.02	1 × 10^−5^
19	**LPA(18:1)**	0.19 ± 0.05	2 × 10^−5^

Differentially decreased lipid list (fold change < 0.5, *p*-value < 0.01); **^a^**, LEV/*L. plantarum*.

## Supplementary Materials

The following are available online at https://www.mdpi.com/1422-0067/21/21/8076/s1.
