# Supplementary material for: Comparative Lipidomic Analysis of Extracellular Vesicles Derived from Lactobacillus plantarum APsulloc 331261 Living in Green Tea Leaves Using Liquid Chromatography-Mass Spectrometry
Source: Int J Mol Sci. 2020 Oct 29;21(21):8076. doi: 10.3390/ijms21218076 (PMC7663264; doi:10.3390/ijms21218076)
Supplement: Supplementary file 1 [file ijms-21-08076-s001.pdf]

Supplementary Materials

Comparative lipidomic analysis of extracellular vesicles derived from *Lactobacillus plantarum* APsulloc 331261 living in green tea leaves using liquid chromatography-mass spectrometry

Hyoseon Kim, Minjung Kim, Kilsun Myoung, Wanil Kim, Jaeyoung Ko, Kwang Pyo Kim, and Eun-Gyung Cho

Supplementary Figures

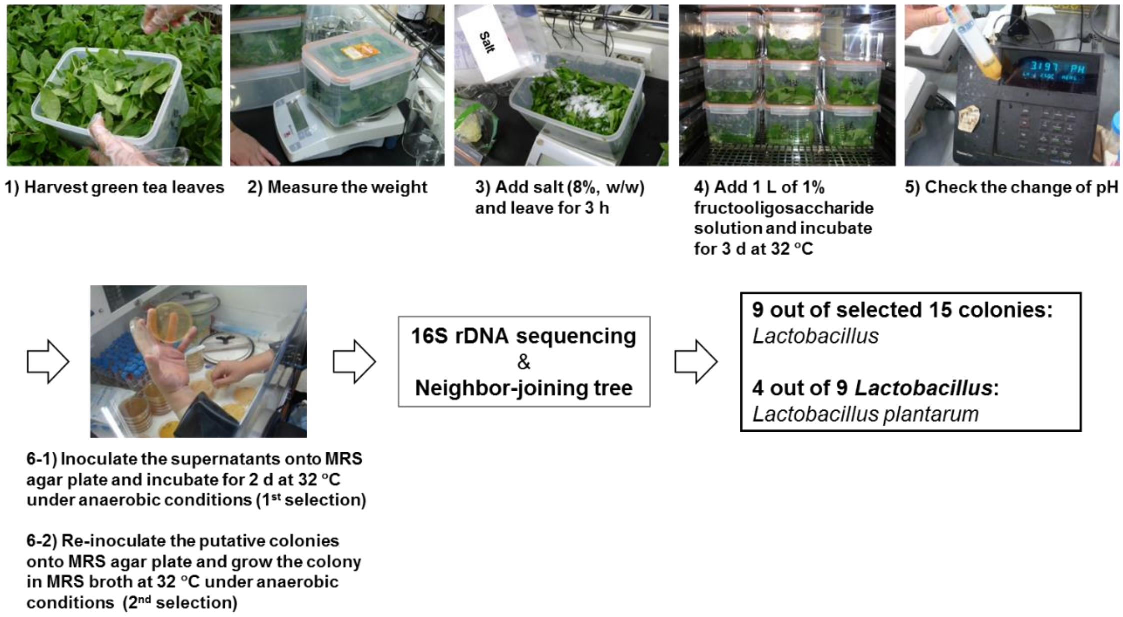

Supplementary Figure S1. Process of isolation of lactic acid bacteria from green tea leaves. Schematic flow for the isolation and validation of LAB from green tea leaves shown.

A **16S rDNA partial sequencing**

> *APsulloc* 331261 (AP1)

TCATGCACGTCGACGACTCTGGTATTGATTGGAGCTTGCATCATGATTTACATTTGAGTGAGTGGCGAACTGGTGAGTAA
CACGTGGGAAACCTGCCCAGAAGCGGGGGATAACACCTGGAAACAGATGCTAATACCGCATAACAACTTGGACCGCAT
GGTCCGAGTTTGAAAGATGGCTTCGGCTATCACTTTTGGATGGTCCCGCGGCGTATTAGCTAGATGGTGGGGTAACGGC
TCACCATGGCAATGATACGTAGCCGACCTGAGAGGGTAATCGGCCACATTGGGACTGAGACACGGCCCAAACTCCTAC
GGGAGGCAGCAGTAGGGAATCTTCCACAATGGACGAAAGTCTGATGGAGCAACGCCGCGTGAGTGAAGAAGGGTTTCG
GCTCGTAAAACTCTGTTGTTAAAGAAGAACATATCTGAGAGTAACTGTTCAGGTATTGACGGTATTTAACCAGAAAGCCA
CGGCTAACTACGTGCCAGCAGCCGCGGTAATACGTAGGTGGCAAGCGTTGTCCGGATTTATTGGGCGTAAAGCGAGCG
CAGGCGGTTTTTTAAGTCTGATGTGAAAGCCTTCGGCTCAACCGAAGAAGTGCATCGGANACTGGGAAACTTGAGTGCA
GAAGAGGACAGTGGAACTCCATGTGTAGCGGTGAAATGCGTAGATATATGGAAGAACACCAGTGGCGAAGGCGGCTGT
CTGGTCTGTAACTGACGCTGAGGCTCGAAAGTATGGGTAGCAAACAGGATTAGATACCCTGGTAGTCCATACCGTAAAC
GATGAATGCTAAGTGTTGGAGGGTTTCCGCCCTTCAGTGCTGCAGCTAACGCATTAAGCATTCCGCCTGGGGAGTACGG
CCGCAAGGCTGAAACTCAAAGGAATTGACGGGGGCCCGCACAAGCGGTGGAGCATGTGGTT

B

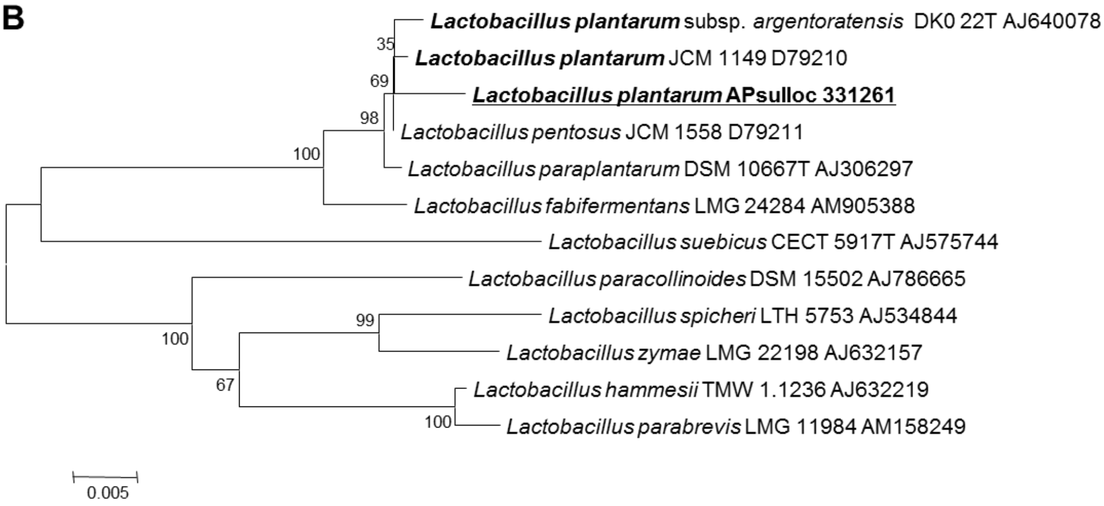

Supplementary Figure S2. Identification of green tea leaf-derived lactic acid bacteria. (A) 16S rDNA partial sequencing result of APsulloc 331261 (AP1). (B) The analysis of neighbor-joining tree based on partial 16S rDNA sequence of AP1. Nine of 15 LAB colonies were the genus *Lactobacillus* and four of them including AP1 were the species *L. plantarum*.

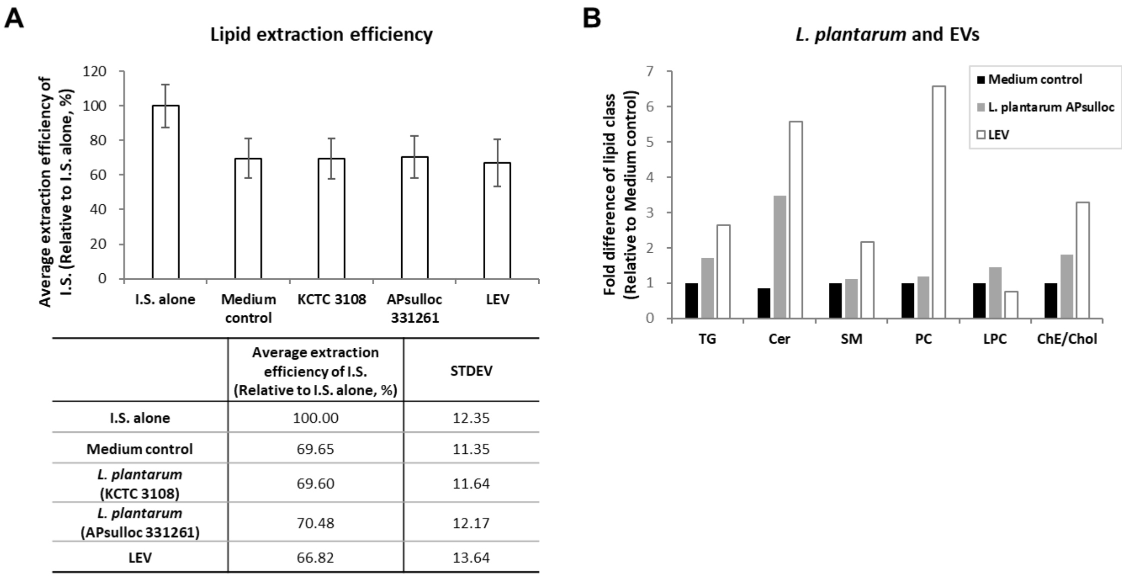

	Average extraction efficiency of I.S. (Relative to I.S. alone, %)	STDEV
I.S. alone	100.00	12.35
Medium control	69.65	11.35
L. plantarum (KCTC 3108)	69.60	11.64
L. plantarum (APsulloc 331261)	70.48	12.17
LEV	66.82	13.64

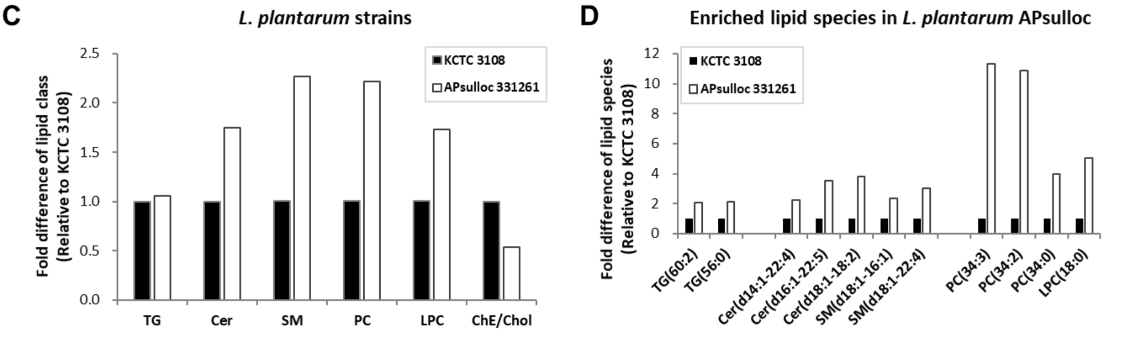

Supplementary Figure S3. Extraction efficiency and relative expression of lipids in the medium control, LEV, and *L. plantarum* strains. (A) Lipid extraction efficiency among the groups. The data is shown as mean ± S.D. of the average extraction efficiency of internal standards (I.S.) of the sample groups relative to that of IS alone in solvent (n = 3 per group; three technical replicates). STDEV, standard deviation. (B, C) The value of fold difference of lipid classes is shown as an average of fold difference of all respective lipid species in LEVs and *L. plantarum* APsulloc 331261 relative to the medium control (B) and in *L. plantarum* APsulloc 331261 relative to *L. plantarum* KCTC 3108 (C). (D) Lipid species showing the increase more than two folds in *L. plantarum* APsulloc 331261 compared to *L. plantarum* KCTC 3108 were displayed by class.

Heat map of identified lipids

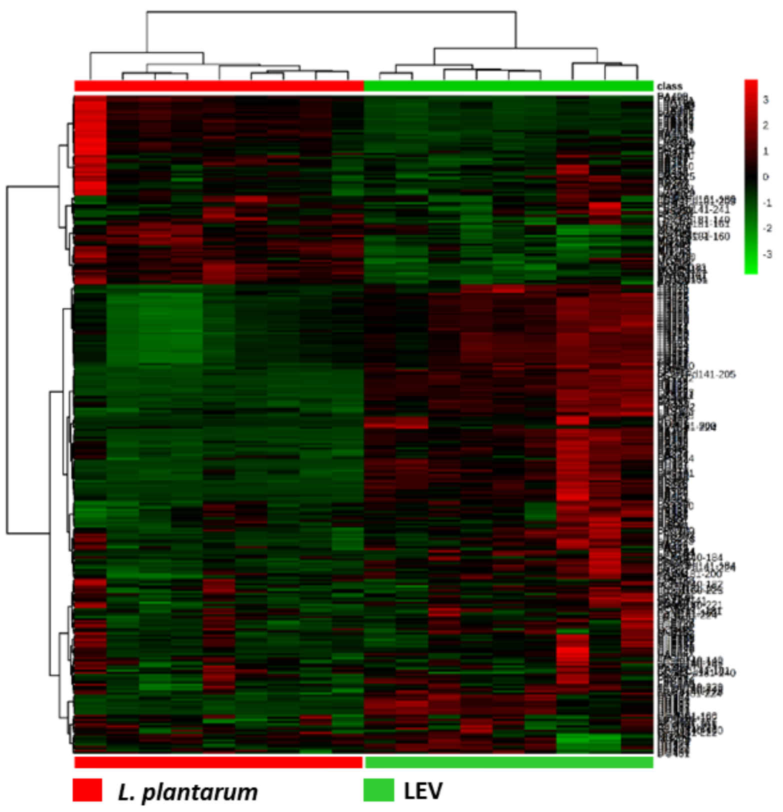

Supplementary Figure S4. Hierarchical clustering of total identified lipids in LEVs versus *L. plantarum*. The heat map shows graphically the differential expression of lipids between LEVs compared to *L. plantarum*. The relative difference is encoded by color intensity. Red: increased; Green: decreased.

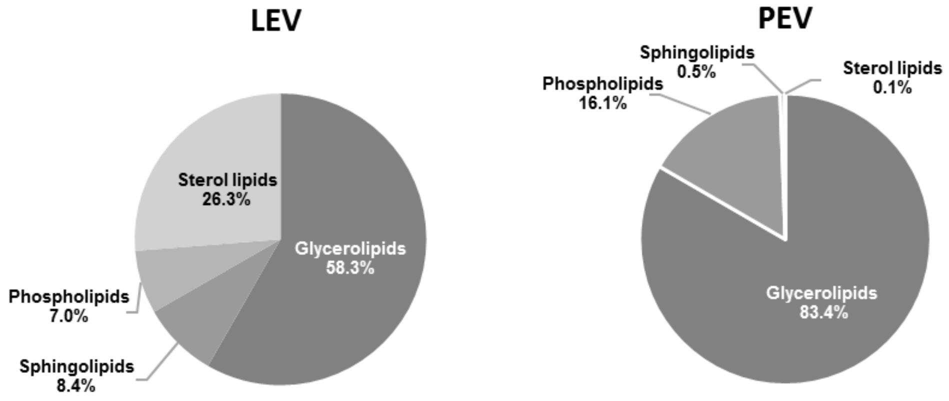

Supplementary Figure S5. Pie diagram for lipid composition in LEVs and PEVs. Composition and proportion of lipids expressed as a percentage of the total lipid amount (mol%).

Supplementary Tables

Table S1. Optimized MRM conditions for targeted lipid classes				
**Lipids**	**Ion mode**	**MRM transition**	**MS/MS CE (eV)**	**Declustering potential**
**MG**	Positive	$[M+NH_4]>[M+NH_4–35]$	20	70
**DG**	Positive	$[M+NH_4]>[M+NH_4–35]$	20	100
**TG**	Positive	$[M+NH_4]>[M+NH_4–RCOONH_4]$	30	90
**ChE**	Positive	$[M+NH_4]>369$	20	50
**Cholesterol**	Positive	$[M+NH_4]>369$	20	90
**PC**	Positive	[M+H]>184	30	135
**PE**	Positive	[M+H]>[M+H–141]	20	90
**PG**	Positive	$[M+NH_4]>[M+NH_4–189]$	20	110
**LPC**	Positive	[M+H]>184	30	90
**LPE**	Positive	[M+H]>[M+H–141]	20	60
**LPG**	Positive	[M+H]>[M+H–172]	20	100
**SM**	Positive	[M+H]>184	30	120
**Cer**	Positive	[M+H]>264	30	90
**dCer**	Positive	[M+H]>266	40	70
**dSM**	Positive	[M+H]>184	50	120
**Methylated PS**	Positive	[M+H]>[M+H–213]	30	70
**Methylated PI**	Positive	[M+H]>[M+H–274]	20	100
**Methylated PA**	Positive	[M+H]>[M+H–126]	20	100
**Methylated LPS**	Positive	[M+H]>[M+H–213]	30	50
**Methylated LPI**	Positive	[M+H]>[M+H–274]	20	110
**Methylated LPA**	Positive	[M+H]>[M+H–126]	20	110
**Methylated Cer1P**	Positive	[M+H]>264	40	70
**Methylated SO1P**	Positive	[M+H]>264	20	60
**Methylated SA1P**	Positive	[M+H]>266	30	90
**Methylated dCer1P**	Positive	[M+H]>266	60	110

Supplementary Table S1. Optimized MRM conditions for targeted lipid classes. All lipid classes analyzed were detected in positive ion mode. Glycerolipids, sterol lipids, and PG were detected as $[M + NH_4]+$ ions. Other phospholipids and sphingolipids were detected as [M + H]+ ions. MS/MS collision energy (CV) was optimized for each lipid transition.

Table S2. Criteria for the quantification of lipids based on the MRM analyses and LODs of internal lipid standards					
**Lipids[a)]**	**RT[b)] (min)**	**Correlation (R^2)**	**Linear range (pg)**	**LOD (pg)**	**Peak area %CV[c)]**
**MG**	1.4	0.9939	0.5-4000	0.5	2.8
**DG**	1.27	0.9987	0.5-4000	0.5	4.5
**TG**	3.58	0.994	0.02-160	0.02	5.4
**ChE**	14.18	0.9972	0.5-4000	0.5	9.5
**PC**	1.5	0.9941	0.02-160	0.02	3.2
**PE**	1.48	0.9963	0.1-800	0.1	6.8
**PG**	1.42	0.9918	0.05-400	0.05	2.1
**LPC**	1.2	0.9983	0.02-160	0.02	**17.6**
**LPE**	1.13	0.9941	0.1-800	0.1	13.4
**LPG**	1.07	0.9844	0.1-800	0.1	3.0
**SM**	2.87	**0.9834**	0.05-400	0.05	3.7
**Cer**	3.07	0.9905	0.1-800	0.1	5.9
**dCer**	3.33	0.9839	0.1-800	0.1	3.2
**dSM**	3.10	0.9925	0.01-80	0.01	4.1
**Methylated PS**	1.61	0.985	0.05-1500	0.05	2.1
**Methylated PI**	1.13	0.9973	0.5-750	0.5	1.6
**Methylated PA**	2.14	0.9932	0.1-400	0.1	2.0
**Methylated LPS**	1.61	0.9999	0.1-1500	0.1	6.9
**Methylated LPI**	1.13	0.9885	0.5-750	0.5	3.3
**Methylated LPA**	2.04	0.9889	0.005-40	0.005	2.9
**Methylated Cer1P**	2.93	0.995	0.05-75	0.05	4.3
**Methylated SO1P**	1.23	0.9871	0.5-750	0.5	4.0
**Methylated SA1P**	1.23	0.9863	0.5-750	0.5	5.6
**Methylated dCer1P**	5.24	0.9983	0.5-750	0.5	15.3

a) Lipid standards used in this study are as follows: MG (15:1), DG (8:0-8:0), TG (11:1-11:1-11:1), ChE (10:0), PC (10:0-10:0), PE (10:0-10:0), PG (10:0-10:0), LPC (13:0), LPE (14:0), LPG (14:0), SM (d18:1-12:0), Cer (d18:1-12:0), dCer (d18:1-12:0), dSM (d18:1-12:0), PS (10:0-10:0), PI (8:0-8:0), PA (10:0-10:0), LPS (17:1), LPI (13:0), LPA (17:0), Cer1P (d18:1-12:0), SO1P (d17:1), SA1P (d17:0), and dCer1p (d18:0-16:0); **b) Retention time**; **c) Coefficient of variation**

Supplementary Table S2. Criteria for the quantification of lipids based on the MRM analyses and LODs of internal lipid standards. To quantify lipid species, respective lipid standard was analyzed three times based on the MRM method. The expression of target lipid was quantified based on the retention time (RT), linear range, and limit of detection (LOD) of respective lipid standard. The coefficient of variation (CV) of the peak area of lipid standards was calculated to be 17.6 or less.

EV	Purification	Protein Conc. (mg/ml)[1)]	Particle No. (per ml)[2)]	Particle No. (per mg protein)
LEV	OptiPrep gradient 1	0.4	2.95E+10	**7.38E+10**
LEV	OptiPrep gradient 2	1.25	5.75E+10	**4.60E+10**
LEV	OptiPrep gradient 3	0.14	5.50E+10	**3.93E+11**

[1)] A Bradford protein assay; [2)] TRPS analysis by qNano-Gold

Supplementary Table S3. Quantification of particle numbers of LEVs. The particle numbers of LEVs purified by density gradient ultracentrifugation (OptiPrep gradient, 1 to 3) or conventional ultracentrifugation after tangential flow filtration were determined using a tunable resistive pulse sensing method (qNano-Gold). Total proteins were quantified using a Bradford protein assay.

No.	Lipid species	LEV (Mean ± S.D.)	*L. plantarum* (Mean ± S.D.)
1	**PE(30:0)**	6.98±4.571	N/A
2	**DG(42:8)**	5.104±3.25	N/A
3	**PE(32:3)**	4.223±2.916	N/A
4	**LPE(22:0)**	2.435±1.985	N/A
5	**LPC(22:4)**	1.918±0.874	N/A
6	**LPE(22:3)**	1.834±0.995	N/A
7	**PE(32:2)**	1.769±1.457	N/A
8	**LPE(22:2)**	0.78±0.467	N/A
9	**DG(38:4)**	N/A	17.34±5.303
10	**dCer1P(d14:1-16:0)**	N/A	0.137±0.066

[nmol/mg protein]

Supplementary Table S4. Uncommon lipid species detected in either LEVs or *L. plantarum*. Ten lipid species were detected only in LEVs (eight) or *L. plantarum* (two). N/A, not available.

Lipid category	Lipid class	LEV (Mean ± S.D.)[a]	*L.plantarum* (Mean ± S.D.)[b]
**Glycerolipids**	**MG**	1071.86 ± 246.55	2104.87 ± 140.41
	**DG**	3400.95 ± 1109.25	1632.66 ± 195.72
	**TG**	3889.5 ± 729.00	1884.19 ± 709.99
**Sphingolipids**	**Cer**	38.61 ± 5.28	37.23 ± 5.55
	**dCer**	903.06 ± 174.95	688.77 ± 227.93
	**SM**	8.34 ± 1.47	6.95 ± 0.37
	**dSM**	47.16 ± 5.06	41.23 ± 6.19
	**Cer1P**	1.41 ± 0.19	1.55 ± 0.14
	**SA1P**	193.18 ± 53.49	278.12 ± 45.82
	**SO1P**	11.84 ± 3.18	21.8 ± 3.19
	**dCer1P**	6.34 ± 4.51	8.79 ± 5.66
**Glycero-phospholipids**	**PA**	9.87 ± 0.54	9.06 ± 0.25
	**PC**	271.24 ± 163.05	37.14 ± 15.31
	**PG**	10.78 ± 8.02	17.06 ± 9.57
	**LPA**	6.98 ± 1.29	14.98 ± 5.99
	**LPC**	272.68 ± 85.46	173.84 ± 74.63
	**LPE**	88.94 ± 40.41	72.43 ± 33.09
	**LPG**	5.78 ± 2.01	7.36 ± 3.42
	**PI**	1.95 ± 0.3	1.28 ± 0.1
	**LPI**	11.98 ± 3.28	6.76 ± 1.63
	**PS**	18.71 ± 3.88	8.44 ± 1.53
	**LPS**	308.18 ± 155.77	132.33 ± 19.53
**Sterol lipids**	**Cholesterol**	60.55 ± 29.37	104.86 ± 25.25
	**ChE**	3707.19 ± 1139.01	2981.42 ± 1103.68

[a, b], [nmol/mg protein]

Supplementary Table S5. Summary for the quantitative value of lipid class in LEVs and *L. plantarum*. The quantitative value of lipid class represents the sum of the amounts of lipid species determined by measuring their peak area using the linear calibration curves established with the internal standards representing a specific lipid family. The data are shown as mean ± S.D. (nmol/mg protein; n = 9 per group with three independent biological replicates & three technical replicates).

(A) Phospholipids

Species	LEV	Cell
PA(48:0)	51.61 ± 0.86	55.41 ± 1.86
PA(46:2)	73.15 ± 9.81	52.43 ± 0.89
PA(46:1)	55.39 ± 2.69	52.33 ± 0.63
PA(46:0)	53.1 ± 2.09	54.05 ± 1.68
PA(44:10)	51.9 ± 0.81	52.31 ± 0.85
PA(44:9)	53.21 ± 1.15	52.32 ± 0.73
PA(44:5)	52.4 ± 0.6	52.18 ± 0.69
PA(44:1)	52.98 ± 0.89	53.06 ± 1.08
PA(42:9)	51.99 ± 0.45	52.57 ± 0.86
PA(42:8)	77.82 ± 13.95	52.59 ± 0.63
PA(42:7)	55.95 ± 2.05	52.28 ± 1.05
PA(42:6)	53.98 ± 1.53	53.35 ± 2.35
PA(42:1)	57.1 ± 3.63	52.2 ± 0.8
PA(40:10)	52.77 ± 1.38	52.85 ± 1.31
PA(40:9)	51.71 ± 0.61	53.64 ± 1.35
PA(40:6)	54.38 ± 2.04	52.37 ± 0.99
PA(40:5)	55.25 ± 1.83	52.5 ± 1.03
PA(40:4)	52.96 ± 0.87	52.98 ± 1.91
PA(40:3)	54.32 ± 1.07	52.25 ± 0.62
PA(40:2)	52.1 ± 0.62	52.26 ± 0.66
PA(40:1)	68.5 ± 6.62	52.07 ± 0.99
PA(38:2)	51.63 ± 0.58	52.85 ± 1.28
PA(38:1)	51.74 ± 0.67	52.73 ± 1.21
PA(38:0)	55.22 ± 1.44	52.54 ± 0.79
PA(36:8)	59.56 ± 2.72	53.16 ± 1.34
PA(36:7)	**100 ± 17.72**	52.98 ± 1.24
PA(36:6)	60.44 ± 3.62	52.79 ± 1.65
PA(36:5)	55.96 ± 3.17	53.63 ± 1.5
PA(36:4)	55.84 ± 2.77	53.25 ± 1.02
PA(36:3)	57.81 ± 2.49	55.95 ± 1.45
PA(36:1)	51.51 ± 0.54	52.29 ± 1
PA(36:0)	74.84 ± 11.99	52.38 ± 0.79
PA(34:6)	53 ± 1.07	53.67 ± 1.82
PA(34:5)	59.58 ± 2.28	54.55 ± 1.79
PA(34:2)	53.99 ± 1.8	54.02 ± 2.41
PA(32:6)	99.55 ± 25.29	55.75 ± 1.88
PA(32:5)	61.09 ± 4.17	56.01 ± 2.86
PA(32:4)	52.35 ± 0.95	55.21 ± 2.29
PA(32:2)	63.83 ± 4.46	55.96 ± 2.55
PA(32:1)	57.74 ± 2.12	53.96 ± 1.22
PA(32:0)	54.87 ± 1.2	54.84 ± 1.26
PA(30:6)	53.07 ± 0.6	57.6 ± 3.15
PA(30:5)	54.45 ± 1.29	57.52 ± 3.06
PA(30:4)	70.76 ± 11.27	56.28 ± 1.79
PA(30:3)	86.27 ± 11.81	57.75 ± 3.63
PA(30:2)	57.76 ± 2.12	57.88 ± 3.78
PA(30:1)	56.36 ± 1.92	57.12 ± 2.57
PA(28:2)	53.14 ± 1.05	58.96 ± 3.65
PA(28:1)	56.1 ± 1.37	59.48 ± 3.2

Relative percentage ± S.D

Species	LEV	Cell
PC(38:7)	0.88 ± 0.15	0.74 ± 0.09
PC(38:5)	2.08 ± 0.52	0.8 ± 0.16
PC(38:4)	2.39 ± 0.67	0.75 ± 0.1
PC(36:5)	1.55 ± 0.56	1.22 ± 0.4
PC(36:4)	6.06 ± 1.96	3.86 ± 2.58
PC(36:3)	8.58 ± 5.8	1.49 ± 0.79
PC(36:2)	38.75 ± 43.94	2.07 ± 1.18
PC(36:1)	4.63 ± 4.54	0.72 ± 0.15
PC(34:3)	3.05 ± 0.66	1.43 ± 0.55
PC(34:2)	**100 ± 75.72**	5.3 ± 3.14
PC(34:1)	30.36 ± 11.72	4.82 ± 2.45
PC(34:0)	3.32 ± 1.43	1.64 ± 0.57
PC(32:2)	23.66 ± 10.97	1.09 ± 0.4
PC(32:1)	13.75 ± 5.1	2.34 ± 1.01
PC(32:0)	6.97 ± 1.82	2.4 ± 0.78
PC(30:1)	23.03 ± 7.08	1.33 ± 0.38
PC(30:0)	3.89 ± 1.1	1.25 ± 0.46
PC(28:3)	2 ± 0.42	2.08 ± 0.52
PC(28:1)	2.67 ± 1.02	1.88 ± 0.94
PC(28:0)	1.43 ± 0.35	0.93 ± 0.22

Relative percentage ± S.D

Species	LEV	Cell
PG(40:1)	0.53 ± 0.04	0.46 ± 0.01
PG(38:3)	0.5 ± 0.03	0.52 ± 0.07
PG(36:5)	0.56 ± 0.05	0.49 ± 0.01
PG(34:5)	0.81 ± 0.13	0.53 ± 0.06
PG(32:5)	0.56 ± 0.05	0.66 ± 0.14
PG(32:3)	0.71 ± 0.07	0.78 ± 0.14
PG(30:6)	0.53 ± 0.02	0.53 ± 0.02
PG(30:3)	0.61 ± 0.05	0.67 ± 0.13
PG(30:0)	0.51 ± 0.04	0.59 ± 0.05
PG(28:3)	0.91 ± 0.13	1.14 ± 0.36
PG(28:0)	60.96 ± 50.1	**100 ± 60.17**

Relative percentage ± S.D

Species	LEV	Cell
PS(46:2)	4.64 ± 1.22	2.1 ± 0.05
PS(42:8)	4.87 ± 1.71	2.13 ± 0.1
PS(40:4)	3.48 ± 0.64	2.3 ± 0.13
PS(40:3)	4.59 ± 0.66	2.32 ± 0.11
PS(40:1)	4.21 ± 0.85	3.66 ± 0.51
PS(38:9)	6.65 ± 1.22	5.23 ± 1.04
PS(38:2)	5.14 ± 1.16	4.16 ± 0.61
PS(38:1)	3.09 ± 0.34	2.79 ± 0.22

PS(36:8)	35.81 ± 7.99	6.28 ± 1.21
PS(36:6)	5.51 ± 0.82	2.37 ± 0.22
PS(34:6)	5.86 ± 1.26	2.68 ± 0.2
PS(34:5)	13.12 ± 2.62	3.04 ± 0.25
PS(34:4)	3.65 ± 0.39	2.41 ± 0.17
PS(34:3)	10.25 ± 2.47	8.2 ± 1.79
PS(32:2)	**100 ± 24.17**	13.65 ± 2.77
PS(32:0)	5.95 ± 0.8	2.43 ± 0.22
PS(30:4)	10.59 ± 2.01	8.35 ± 1.95
PS(30:3)	6.65 ± 1.44	4.82 ± 0.89
PS(30:0)	17.02 ± 11.92	20.71 ± 12.37
PS(28:5)	23.48 ± 5.99	19.15 ± 3.77
PS(28:4)	6.81 ± 1.56	5.67 ± 0.92
PS(28:3)	3.56 ± 0.47	3.07 ± 0.24
PS(28:2)	3.8 ± 0.55	2.66 ± 0.17

Relative percentage ± S.D

Species	LEV	Cell
PI(40:5)	39.89 ± 4.69	27.64 ± 1.14
PI(38:3)	33.37 ± 4.17	32.42 ± 1.92
PI(32:6)	**100 ± 30.01**	33.55 ± 4.41
PI(32:5)	51.97 ± 10.44	45.06 ± 5.75
PI(30:6)	41.25 ± 7.6	35.39 ± 4.56
PI(30:3)	69.56 ± 9.77	37 ± 2.83
PI(28:4)	43.16 ± 8.06	37.86 ± 6.32

Relative percentage ± S.D

Species	LEV	Cell
LPA(22:5)	10.67 ± 1.91	15.61 ± 5.38
LPA(22:4)	6.34 ± 0.64	15.08 ± 6.14
LPA(22:3)	18.34 ± 5.67	14.28 ± 4.9
LPA(22:2)	12.71 ± 2.28	13.32 ± 4.27
LPA(22:1)	6.78 ± 0.55	11.66 ± 3.47
LPA(22:0)	13 ± 3.83	33.28 ± 15.17
LPA(20:5)	7.92 ± 1.1	22.94 ± 8.27
LPA(20:3)	7.38 ± 0.75	14.16 ± 5.5
LPA(20:2)	25.02 ± 9.65	15.27 ± 5.92
LPA(20:1)	8.54 ± 1.3	12.16 ± 4.51
LPA(20:0)	10.27 ± 2.03	26.58 ± 9.49
LPA(18:4)	7.19 ± 1.22	23.24 ± 11.4
LPA(18:3)	7.91 ± 1.03	17.64 ± 7.39
LPA(18:2)	8.25 ± 1.12	21.63 ± 7.9
LPA(18:1)	13.25 ± 2.26	69.46 ± 28.12
LPA(16:0)	37.59 ± 10.94	**100 ± 44.3**
LPA(14:0)	21.27 ± 7.99	50.77 ± 26.47

Relative percentage ± S.D

Species	LEV	Cell
LPC(22:6)	**100 ± 32.87**	64.61 ± 27.38
LPC(22:5)	1.68 ± 0.78	0.9 ± 0.46
LPC(22:0)	1.25 ± 0.37	0.94 ± 0.54
LPC(20:0)	0.99 ± 0.64	1.04 ± 0.62
LPC(18:2)	0.69 ± 0.38	0.55 ± 0.44
LPC(18:1)	2 ± 0.88	0.58 ± 0.44
LPC(18:0)	1.75 ± 0.68	0.54 ± 0.32
LPC(16:0)	8.3 ± 3.36	5.19 ± 2.26

Relative percentage ± S.D

Species	LEV	Cell
LPE(22:5)	3.5 ± 1.25	2.27 ± 1.62
LPE(22:6)	**100 ± 46.46**	65.38 ± 35.99

Relative percentage ± S.D

Species	LEV	Cell
LPG(22:5)	4.29 ± 1.39	4.4 ± 1.07
LPG(22:3)	8.08 ± 2.03	5.47 ± 2.14
LPG(22:2)	14.99 ± 4.47	4.67 ± 1.63
LPG(22:1)	3.89 ± 1.2	3.23 ± 0.84
LPG(22:0)	10.94 ± 2.23	12.3 ± 3.89
LPG(20:3)	8.77 ± 1.85	6.42 ± 2.88
LPG(20:2)	8.57 ± 2.68	6.09 ± 2.2
LPG(20:1)	10.46 ± 2.76	10.72 ± 3.61
LPG(18:4)	56.16 ± 54.83	9.81 ± 4.93
LPG(18:3)	42.51 ± 21.28	**100 ± 50.73**
LPG(18:2)	22.79 ± 9.65	61.91 ± 27.87
LPG(18:1)	11.33 ± 3.75	20.47 ± 11.65
LPG(16:0)	11.45 ± 4.83	27.39 ± 16.21

Relative percentage ± S.D

Species	LEV	Cell
LPI(22:5)	10.09 ± 5.04	23.43 ± 6.41
LPI(22:4)	5.88 ± 3.26	11.16 ± 4.12
LPI(22:3)	69.64 ± 20.43	29.33 ± 16.47
LPI(22:2)	**100 ± 30.84**	14.72 ± 4.55
LPI(20:3)	51.89 ± 14.12	50.04 ± 17.72
LPI(20:2)	44.68 ± 14.14	28.77 ± 6.13
LPI(20:1)	15.18 ± 3.46	8.28 ± 2.54
LPI(18:1)	9.1 ± 4.66	8.99 ± 2.79
LPI(16:1)	30.65 ± 8.48	28.05 ± 8.13
LPI(16:0)	61.52 ± 14.76	24.1 ± 4.34
LPI(14:0)	28.73 ± 12.74	14.13 ± 3.78

Relative percentage ± S.D

Species	LEV	Cell
LPS(22:5)	1.68 ± 0.74	1.32 ± 0.5
LPS(22:4)	2.22 ± 0.7	1.38 ± 0.5
LPS(22:3)	15.79 ± 4.71	2.7 ± 0.56
LPS(22:2)	19.95 ± 5.02	3.1 ± 0.42
LPS(22:1)	3.8 ± 1.11	1.05 ± 0.27
LPS(22:0)	13.4 ± 4.45	11.7 ± 3.32
LPS(20:5)	3.47 ± 1.38	1.53 ± 0.37
LPS(20:3)	3.29 ± 0.76	1.84 ± 0.46
LPS(20:0)	6.45 ± 1.5	4.94 ± 1.23
LPS(18:4)	**100 ± 93.79**	4.61 ± 1.54
LPS(18:3)	5.05 ± 2.86	2.23 ± 0.8
LPS(18:1)	11.52 ± 2.97	10.75 ± 2.41
LPS(18:0)	7.82 ± 4.33	9.11 ± 2.02
LPS(16:1)	24.21 ± 8.25	30.23 ± 6.43
LPS(16:0)	6.95 ± 2.18	7.95 ± 1.11
LPS(14:0)	6.86 ± 2.15	5.36 ± 0.98

Relative percentage ± S.D

(B) Sphingolipids

Species	LEV	Cell
Cer(d14:1-16:0)	60.73 ± 25.4	57.16 ± 17.64
Cer(d14:1-22:4)	55.78 ± 21.54	39.21 ± 4.53
Cer(d16:1-16:0)	46.98 ± 8.22	47.17 ± 10.93
Cer(d16:1-22:2)	37.22 ± 4.11	37.15 ± 5.76
Cer(d16:1-22:5)	39.45 ± 6.1	39.74 ± 5.15
Cer(d18:1-18:2)	92.19 ± 29.69	**100 ± 30.17**
Cer(d14:1-22:3)	39.68 ± 7.16	38.4 ± 3.54

Relative percentage ± S.D

Species	LEV	Cell
dCer(d14:0-14:0)	8.95 ± 3.63	8.57 ± 1.91
dCer(d14:0-18:3)	28.12 ± 7.84	25.53 ± 9.27
dCer(d14:0-18:4)	11.93 ± 6.5	10.25 ± 3.05
dCer(d14:0-22:0)	37.94 ± 10.9	28.35 ± 13.53
dCer(d14:0-22:1)	13.26 ± 3.9	10.78 ± 5.8
dCer(d14:0-24:0)	**100 ± 17.96**	63.85 ± 24.16
dCer(d14:0-24:1)	11.55 ± 4.84	10.14 ± 3.48
dCer(d14:0-16:0)	7.31 ± 3.49	6.61 ± 2.7
dCer(d14:0-18:2)	12.27 ± 4.83	13.04 ± 6.59
dCer(d14:0-22:3)	12.91 ± 3.89	8.67 ± 3.28
dCer(d16:0-22:3)	5.6 ± 1.67	4.75 ± 2.48

Relative percentage ± S.D

Species	LEV	Cell
SM(d18:1-16:1)	89.91 ± 10.76	85.27 ± 11.61
SM(d18:1-20:0)	**100 ± 36.82**	66.33 ± 2.98
SM(d18:1-22:4)	70.29 ± 6.84	65.06 ± 2.63

Relative percentage ± S.D

Species	LEV	Cell
dSM(d18:1-18:1)	**100 ± 27.78**	86.57 ± 17.17
dSM(d18:1-18:4)	75.73 ± 16.11	70.78 ± 16
dSM(d18:1-20:0)	74.35 ± 10.22	64.03 ± 11.46
dSM(d18:1-22:4)	78.94 ± 10.33	66.28 ± 12.56

Relative percentage ± S.D

Species	LEV	Cell
Cer1P(d14:1-18:1)	61.53 ± 5.9	62.43 ± 6.37
Cer1P(d14:1-24:1)	93.29 ± 34.42	**100 ± 23.29**
Cer1P(d18:1-14:0)	60.8 ± 7.15	70.57 ± 7.46
Cer1P(d18:1-16:0)	55.79 ± 2.97	66.5 ± 6.12
Cer1P(d18:1-16:1)	88.89 ± 15.05	95.19 ± 13.2

Relative percentage ± S.D

Species	LEV	Cell
SA1P(d14:1)	39.81 ± 11.55	31.42 ± 5.63
SA1P(d16:1)	9.94 ± 3.44	28.7 ± 5.22
SA1P(d18:1)	61.45 ± 18.11	**100 ± 17.53**

Relative percentage ± S.D

Species	LEV	Cell
SO1P(d14:1)	3.86 ± 1.07	7.81 ± 1.37
SO1P(d16:1)	3.91 ± 1.01	6.89 ± 1.49
SO1P(d18:1)	54.56 ± 15.59	**100 ± 15.17**

Relative percentage ± S.D

Species	LEV	Cell
dCer1P(d14:1-18:4)	5.33 ± 1.85	0.92 ± 0.43
dCer1P(d14:1-20:5)	4.12 ± 1.24	0.78 ± 0.47
dCer1P(d14:1-22:4)	3.42 ± 1.44	0.95 ± 0.5
dCer1P(d16:1-18:0)	21.91 ± 17.8	34.32 ± 23.31
dCer1P(d18:1-20:5)	64.06 ± 51.24	**100 ± 64.62**
dCer1P(d18:1-24:0)	0.48 ± 0.33	0.71 ± 0.29

Relative percentage ± S.D

(C) Glycerolipids

Species	LEV	Cell
MG(14:0)	14.19 ± 2.55	20.92 ± 3.6
MG(16:1)	3.05 ± 0.58	5.17 ± 0.57
MG(18:1)	7.79 ± 1.15	16.63 ± 2.92
MG(18:2)	8.05 ± 1.06	10.82 ± 1.49
MG(18:3)	6.18 ± 1.23	10.66 ± 1.43
MG(18:4)	5.5 ± 0.74	9.7 ± 1.29
MG(20:0)	18.99 ± 7.99	35.8 ± 8.76
MG(20:2)	4.45 ± 2.42	3.46 ± 0.7
MG(20:3)	2.49 ± 0.68	3.92 ± 0.59
MG(20:4)	1.63 ± 0.54	2.73 ± 0.56
MG(20:5)	1.08 ± 0.62	1.98 ± 0.37
MG(22:0)	20.62 ± 6.53	**100 ± 37.61**
MG(22:4)	2.09 ± 0.9	2.94 ± 0.65
MG(22:5)	40.02 ± 16.3	64.36 ± 15.1
MG(22:6)	16.37 ± 6.41	16.96 ± 1.29
MG(24:0)	1.14 ± 0.6	4.12 ± 1.44
MG(24:1)	11.07 ± 2.97	13.35 ± 1.59

Relative percentage ± S.D

Species	LEV	Cell
DG(30:0)	14.97 ± 4.11	23.45 ± 2.9
DG(30:4)	9.62 ± 3.25	10.47 ± 1.56
DG(30:5)	3.43 ± 0.72	4.87 ± 0.75
DG(32:1)	14.96 ± 4.4	12.05 ± 1.98
DG(32:2)	9.2 ± 3.06	4.28 ± 0.72
DG(32:4)	4.04 ± 1.46	4.53 ± 0.63
DG(34:1)	**100 ± 33.52**	23.97 ± 4.39
DG(34:2)	50.71 ± 16.88	14.64 ± 2.43
DG(34:3)	6.97 ± 2.4	2.5 ± 0.79
DG(36:2)	66.6 ± 21.93	11.02 ± 2.51
DG(36:3)	27.25 ± 11.01	6.32 ± 0.8
DG(36:4)	3.65 ± 1.14	1.76 ± 0.36
DG(36:6)	2.58 ± 0.78	1.14 ± 0.28
DG(36:7)	5.57 ± 1.51	4.93 ± 0.7
DG(38:0)	22.38 ± 12.96	31.21 ± 6.1
DG(38:1)	8.19 ± 3.32	2.81 ± 0.64
DG(38:2)	88.23 ± 31.65	35.13 ± 4.93
DG(40:4)	4.64 ± 2.44	8.28 ± 1.51
DG(42:9)	2.41 ± 1.15	1.7 ± 0.42
DG(42:10)	1.35 ± 0.33	2.75 ± 0.75
DG(44:0)	6.59 ± 1.56	6.92 ± 1.72
DG(46:0)	2.92 ± 0.83	2.96 ± 0.76
DG(48:1)	3.15 ± 1.06	2.81 ± 0.99

Relative percentage ± S.D

Species	LEV	Cell
TG(44:0)	21.97 ± 4.1	11.5 ± 3.62
TG(46:1)	23.26 ± 4.26	12 ± 4.13
TG(46:0)	28.62 ± 5.36	14.26 ± 5.3
TG(48:2)	26.96 ± 4.93	13.43 ± 4.84
TG(48:1)	87.27 ± 16.9	42.43 ± 16.29
TG(48:0)	**100 ± 20.18**	51.49 ± 20.2
TG(50:8)	1.96 ± 0.33	1.22 ± 0.48
TG(50:7)	1.14 ± 0.23	0.65 ± 0.22
TG(50:3)	25.15 ± 4.78	11.85 ± 4.63
TG(50:2)	81.36 ± 14.56	39.13 ± 15.43
TG(50:1)	90.83 ± 17.82	44.05 ± 16.85
TG(50:0)	25.04 ± 5.28	12.04 ± 5.18
TG(52:8)	0.46 ± 0.06	0.27 ± 0.07
TG(52:7)	3.84 ± 0.89	1.97 ± 0.63
TG(52:6)	0.38 ± 0.06	0.23 ± 0.05
TG(52:5)	0.56 ± 0.31	0.15 ± 0.04
TG(52:4)	6.2 ± 1.81	2.53 ± 1.03
TG(52:3)	25.66 ± 5.18	11.64 ± 4.45
TG(52:2)	40.77 ± 8.12	19.24 ± 7.6
TG(52:1)	16.66 ± 3.72	7.44 ± 2.64
TG(52:0)	9.42 ± 2.01	4.32 ± 1.61
TG(54:8)	1.87 ± 0.37	0.97 ± 0.31
TG(54:6)	2.01 ± 1.04	0.71 ± 0.36
TG(54:5)	7.79 ± 2.31	3.11 ± 1.07
TG(54:4)	12.43 ± 2.5	5.75 ± 2.16
TG(54:3)	49.08 ± 10.17	21.69 ± 8.13
TG(54:2)	16.01 ± 3.05	7.45 ± 3
TG(54:1)	6.91 ± 1.61	3.26 ± 1.21
TG(54:0)	7.33 ± 1.34	3.82 ± 1.42
TG(56:10)	0.29 ± 0.05	0.16 ± 0.02
TG(56:9)	0.89 ± 0.19	0.48 ± 0.16
TG(56:8)	2.95 ± 0.54	1.49 ± 0.58
TG(56:5)	0.26 ± 0.04	0.14 ± 0.02
TG(56:4)	0.42 ± 0.07	0.21 ± 0.06
TG(56:3)	1.81 ± 0.36	0.79 ± 0.26
TG(56:2)	3.82 ± 0.64	1.77 ± 0.65
TG(56:1)	2.4 ± 0.43	1.27 ± 0.47
TG(56:0)	1.94 ± 0.37	1.06 ± 0.32
TG(58:9)	0.27 ± 0.03	0.18 ± 0.03
TG(58:2)	0.57 ± 0.08	0.34 ± 0.08
TG(58:1)	0.47 ± 0.07	0.3 ± 0.08
TG(58:0)	0.23 ± 0.02	0.18 ± 0.04
TG(60:2)	0.14 ± 0.02	0.12 ± 0.01

Relative percentage ± S.D

(D) Sterol lipids

Species	LEV	Cell
CE(14:0)	12.69 ± 3.37	11.26 ± 3.68
CE(16:0)	20.62 ± 5.41	20.07 ± 8.34
CE(16:1)	**100 ± 25.15**	75.45 ± 25.48
CE(18:0)	4.77 ± 2.7	4.1 ± 1.67
CE(18:1)	49.48 ± 19.52	40.73 ± 16.35
CE(18:2)	8.55 ± 1.56	10.75 ± 3.15
CE(20:0)	6.68 ± 3.9	6.67 ± 3.41
CE(20:1)	5.47 ± 3.62	4.78 ± 2.5
CE(20:2)	3.13 ± 0.95	2.48 ± 0.99
CE(20:3)	2.4 ± 0.74	2.1 ± 0.83
CE(22:0)	7.9 ± 4.94	4.8 ± 2.04
ChE(22:1)	4.33 ± 2.31	3.19 ± 1.34
CE(22:2)	1.34 ± 0.66	1.02 ± 0.5
CE(22:3)	1.85 ± 0.46	1.45 ± 0.41
CE(24:0)	2.97 ± 2.69	1.76 ± 0.82
CE(24:1)	14.03 ± 8.74	7.39 ± 2.75
Cholesterol	4.02 ± 1.95	6.96 ± 1.67

Relative percentage ± S.D

Supplementary Table S6. Relative expression of lipid species in LEVs and *L. plantarum*. The relative expression of lipid species shown in tabular format by class. The identified lipid species were grouped in phospholipids (A), sphingolipids (B), glycerolipids (C), or sterol lipids (D). Boxed tables indicate lipid classes showing overall decreased expression of identified lipid species in LEVs relative to *L. plantarum*. The data are expressed as a percentage relative to the highest expression value (100%) in the class ± S.D. of nine samples from three biological replicates and three technical repeats.

No.	Lipid	LEV (Mean ± S.D.)[a]	*L. plantarum* (Mean ± S.D.)[b]
1	**LPS(18:4)**	132.52±124.3	6.11±2.04
2	**PC(32:2)**	22.99±10.66	1.06±0.39
3	**PC(34:2)**	97.18±73.59	5.15±3.05
4	**PC(30:1)**	22.38±6.88	1.3±0.37
5	**PS(32:2)**	6.48±1.56	0.88±0.18
6	**LPI(22:2)**	2.8±0.86	0.41±0.12
7	**LPS(22:2)**	26.44±6.66	4.1±0.56
8	**PC(34:1)**	29.5±11.39	4.68±2.38
9	**DG(36:2)**	492.98±162.37	81.56±18.59
10	**PC(32:1)**	13.36±4.96	2.28±0.98
11	**LPS(22:3)**	20.93±6.24	3.58±0.75
12	**PC(36:3)**	8.34±5.63	1.44±0.77
13	**dCer1P(d14:1-18:4)**	0.34±0.11	0.05±0.02
14	**PS(36:8)**	2.32±0.51	0.4±0.07
15	**dCer1P(d14:1-20:5)**	0.26±0.07	0.05±0.03
16	**DG(36:3)**	201.74±81.54	46.81±5.97
17	**PS(34:5)**	0.85±0.17	0.19±0.01
18	**DG(34:1)**	740.11±248.15	177.41±32.51
19	**TG(52:5)**	2.98±1.64	0.81±0.23
20	**LPS(22:1)**	5.04±1.48	1.39±0.35
21	**dCer1P(d14:1-22:4)**	0.21±0.09	0.06±0.03
22	**DG(34:2)**	375.31±124.95	108.42±18
23	**LPC(18:1)**	4.68±2.07	1.37±1.03
24	**LPC(18:0)**	4.09±1.61	1.26±0.75
25	**LPG(22:2)**	0.4±0.12	0.12±0.04
26	**PC(38:4)**	2.32±0.65	0.73±0.09
27	**PC(30:0)**	3.78±1.07	1.21±0.45
28	**PI(32:6)**	0.51±0.15	0.17±0.02
29	**DG(38:1)**	60.62±24.59	20.8±4.78
30	**PC(32:0)**	6.77±1.77	2.33±0.76
31	**TG(54:6)**	10.61±5.53	3.78±1.94
32	**DG(34:3)**	51.6±17.82	18.51±5.84
33	**PC(38:5)**	2.02±0.51	0.78±0.16
34	**LPI(16:0)**	1.72±0.41	0.67±0.12
35	**DG(38:2)**	653±234.31	260.01±36.51
36	**TG(54:5)**	41.11±12.19	16.41±5.66
37	**PS(32:0)**	0.38±0.05	0.15±0.01
38	**TG(52:4)**	32.69±9.58	13.39±5.46
39	**LPI(22:3)**	1.95±0.57	0.82±0.46
40	**PS(36:6)**	0.35±0.05	0.15±0.01
41	**PS(42:8)**	0.31±0.11	0.13±0
42	**TG(56:3)**	9.58±1.9	4.21±1.38
43	**LPS(20:5)**	4.6±1.83	2.03±0.49
44	**DG(36:6)**	19.1±5.78	8.43±2.1
45	**TG(54:3)**	258.83±53.66	114.37±42.91
46	**TG(52:1)**	87.85±19.65	39.23±13.92
47	**TG(52:3)**	135.33±27.33	61.41±23.49
48	**PS(46:2)**	0.3±0.07	0.13±0
49	**PS(34:6)**	0.38±0.08	0.17±0.01
50	**TG(52:0)**	49.72±10.64	22.78±8.51

51	**TG(54:4)**	65.56±13.18	30.36±11.43
52	**TG(56:2)**	20.19±3.42	9.35±3.42
53	**DG(32:2)**	68.14±22.7	31.69±5.35
54	**TG(54:2)**	84.46±16.1	39.31±15.85
55	**PC(34:3)**	2.96±0.64	1.39±0.54
56	**TG(50:3)**	132.63±25.22	62.49±24.44
57	**TG(54:1)**	36.45±8.53	17.19±6.41
58	**TG(52:2)**	215.02±42.86	101.49±40.1
59	**TG(50:2)**	429.02±76.8	206.38±81.41
60	**TG(50:0)**	132.07±27.86	63.53±27.32
61	**DG(36:4)**	27.07±8.48	13.06±2.67
62	**TG(50:1)**	478.95±93.98	232.32±88.89
63	**TG(48:1)**	460.19±89.15	223.77±85.91
64	**LPI(14:0)**	0.8±0.35	0.39±0.1
65	**PC(34:0)**	3.22±1.39	1.59±0.55
66	**TG(46:0)**	150.94±28.28	75.21±27.98
67	**TG(48:2)**	142.16±26.01	70.86±25.53

[a, b], [nmol/mg protein]

Supplementary Table S7. Summary for the quantitative value of differentially increased lipid species in LEVs. The quantitative values of differentially increased lipid species in LEVs were determined by measuring their peak area using the linear calibration curves established with the internal standards representing a specific lipid family. The data are shown as mean ± S.D. (nmol/mg protein; n = 9 per group with three independent biological replicates & three technical replicates).

No.	Lipid	LEV (Mean ± S.D.)[a]	*L. plantarum* (Mean ± S.D.)[b]
1	**SO1P(d14:1)**	0.73±0.2	1.48±0.26
2	**DG(42:10)**	10.04±2.5	20.38±5.58
3	**MG(18:1)**	50.67±7.52	108.2±19.04
4	**LPA(18:3)**	0.24±0.03	0.55±0.23
5	**LPI(22:5)**	0.28±0.14	0.65±0.17
6	**LPG(18:3)**	1.14±0.57	2.69±1.36
7	**LPA(22:4)**	0.19±0.02	0.47±0.19
8	**LPA(14:0)**	0.66±0.25	1.59±0.83
9	**LPA(22:0)**	0.4±0.12	1.04±0.47
10	**LPA(20:0)**	0.32±0.06	0.83±0.29
11	**LPA(18:2)**	0.25±0.03	0.67±0.24
12	**LPA(16:0)**	1.18±0.34	3.14±1.39
13	**LPG(18:2)**	0.61±0.26	1.67±0.75
14	**SA1P(d16:1)**	17.27±5.98	49.85±9.08
15	**LPA(20:5)**	0.24±0.03	0.72±0.25
16	**LPA(18:4)**	0.22±0.03	0.72±0.35
17	**MG(24:0)**	7.45±3.94	26.81±9.38
18	**MG(22:0)**	134.16±42.47	650.46±244.65
19	**LPA(18:1)**	0.41±0.07	2.18±0.88

[a, b], [nmol/mg protein]

Supplementary Table S8. Summary for the quantitative value of differentially decreased lipid species in LEVs. The quantitative values of differentially decreased lipid species in LEVs were determined by measuring their peak area using the linear calibration curves established with the internal standards representing a specific lipid family. The data are shown as mean ± S.D. (nmol/mg protein; n = 9 per group with three independent biological replicates & three technical replicates).
